# Low-Temperature All-Solid-State Batteries

**DOI:** 10.1007/s40820-026-02270-0

**Published:** 2026-07-27

**Authors:** Hyojoo Lee, Joo Hyeong Suh, Jaeik Kim, Wei Kong Pang, Konstantin Konstantinov, Junyoung Mun, Taeseup Song, Jung Ho Kim

**Affiliations:** 1 Institute for Superconducting & Electronic Materials (ISEM), Australian Institute for Innovative Materials (AIIM), University of Wollongong, Innovation Campus, Squires Way, North Wollongong, NSW 2500 Australia; 2 School of Advanced Materials Science and Engineering, Sungkyunkwan University, Seobu-ro, Jangan-gu, Suwon-si, Gyeonggi-do 2066 Republic of Korea; 3 Department of Energy Engineering, Hanyang University, Seoul, 04763 Republic of Korea; 4 SKKU Institute of Energy Science and Technology (SIEST), Sungkyunkwan University, 2066, Seobu-ro, Jangan-gu, Suwon-si, Gyeonggi-do 16419 Republic of Korea

**Keywords:** Low-temperature secondary battery, All-solid-state battery, Ion conductivity

## Abstract

Reliable battery operation in sub-zero environments is critical for polar exploration, military missions, and space applications. However, conventional lithium-ion batteries (LIBs) face inherent limitations at low temperatures by intrinsic hurdle of poor ionic mobility in liquid electrolytes. All-solid-state batteries (ASSBs), which replace liquid electrolytes with non-flammable and non-freezing solid electrolytes, are considered promising alternatives because these solid electrolytes provide high ionic conductivity at low temperatures, superior temperature stability, and exceptional safety. ASSBs still face practical limitations at extremely low temperatures due to degradation caused by interfacial side reactions and mechanical instabilities that increase resistance and polarization. Addressing these challenges is critical to realize the advantages of ASSBs and enable their practical deployment in low-temperature applications. This review provides an overview of historical developments, critical challenges, and recent progress in advancing the low-temperature performance of ASSBs. The key components of ASSBs, including solid electrolytes, cathodes, and anodes, are systematically investigated to develop strategies for improving ASSBs at low temperatures. By highlighting future perspectives, we emphasize both the potential and necessity of ASSBs to overcome the intrinsic limitations of LIBs and ensure reliable energy storage in harsh environments.

## Highlights

- All-solid-state batteries (ASSBs) overcome the intrinsic limitations of liquid electrolyte based lithium-ion batteries, such as electrolyte freezing and sluggish ion transport, by utilizing non-freezing solid electrolytes with relatively stable ionic conductivity at sub-zero temperatures.
- Performance degradation in ASSBs under sub-zero conditions arises from interconnected factors, including suppressed Li-ion transport, increased interfacial resistance due to side reactions, and mechanical instability, such as contact loss and microcracking.
- Achieving robust low-temperature performance demands the combined advancement of materials design and system-level engineering.

## Graphical abstract

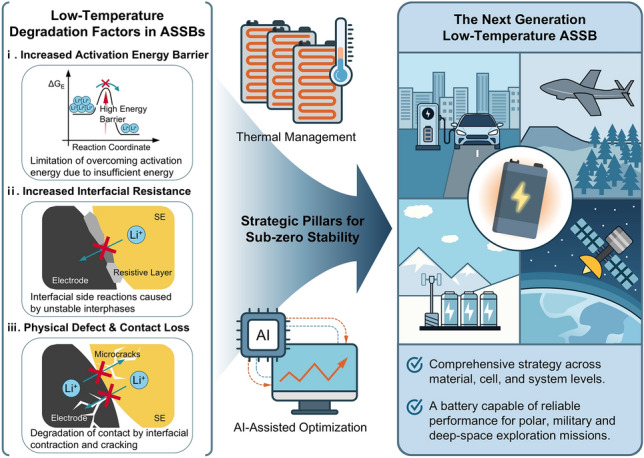

## Introduction

The global demand for secondary batteries has increased significantly in recent years, driven by the rapid growth of electric vehicle (EV) and portable electronic device market [1–7]. With their influence expanding on grid-scale energy storage systems, lithium-ion batteries (LIBs) have become a key technology for enabling a sustainable future, rather than serving as simple power sources [8–13]. Continuous advancements in high-capacity electrode materials, safety-enhanced electrolyte formulations, and optimized cell configurations have led to notable improvements in energy density, cycling stability, and rate capability, gradually expanding their application fields, thereby enabling applications from EVs to aerospace systems operating in sub-zero environments [14–17].

**Fig. 1:**
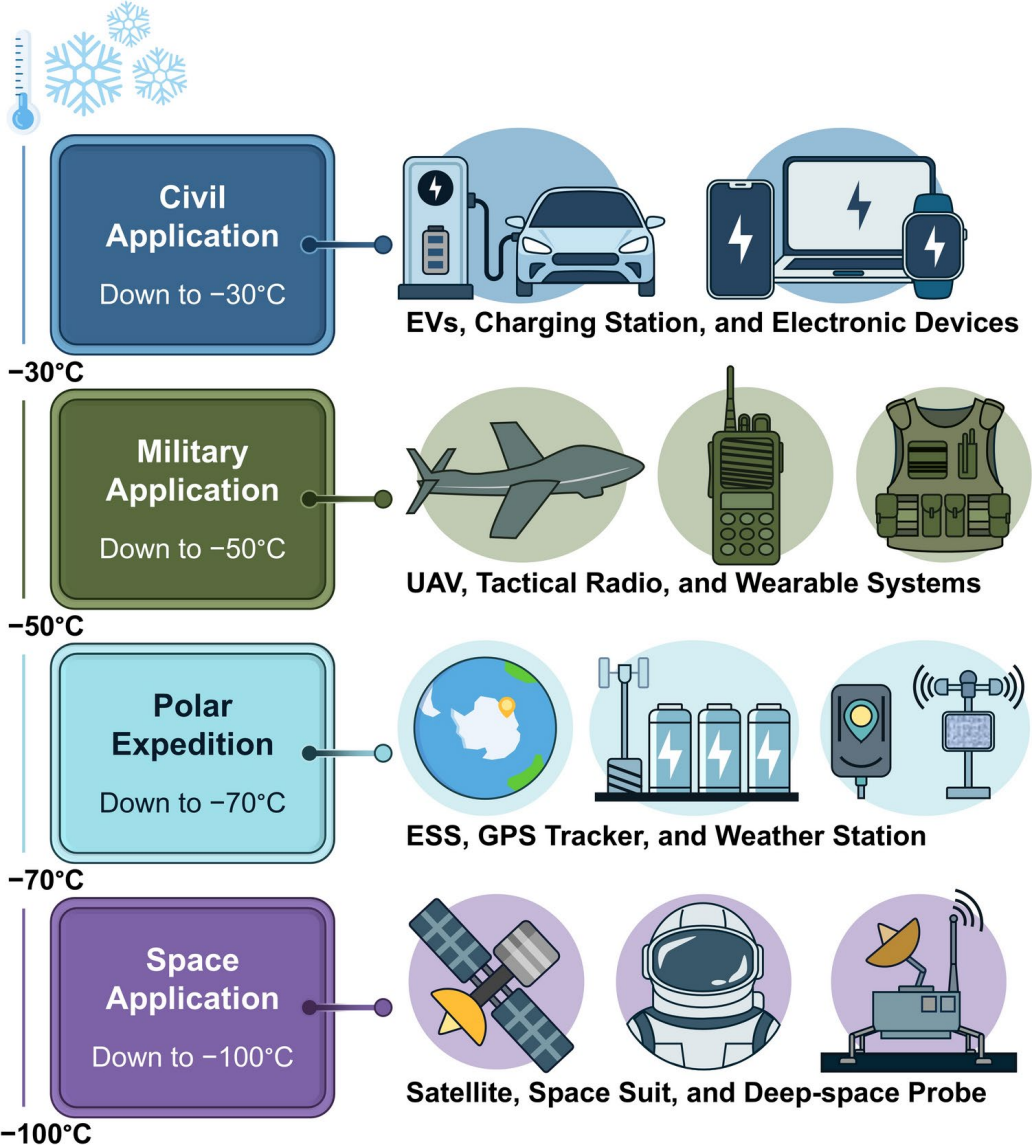
Schematic illustration of low-temperature battery applications classified by operational temperature range: civil applications (down to − 30 °C; electric vehicles (EVs), charging stations, electronic devices), military applications (down to − 50 °C; unmanned aerial vehicles (UAVs), tactical radios, wearable systems), polar expeditions (down to − 70 °C; energy storage systems (ESS), GPS trackers, weather stations), and space applications (down to − 100 °C; satellites, space suits, deep-space probes)

With the growing breadth of application environments, LIBs are increasingly required to operate under harsher conditions [18–22]. As shown in Fig. 1, civil applications such as EVs, charging stations, and portable electronics often face temperatures near − 30 °C [15, 23, 24]. Military systems, including unmanned aerial vehicles (UAVs), tactical radios, and wearable equipments, should be required to function at around − 50 °C. Polar expeditions push the limits further to nearly − 70 °C, where batteries are essential for energy storage systems (ESS), GPS trackers, weather stations, and other field devices. The most severe conditions occur in space exploration, where satellites, space suits, and deep-space probes must withstand temperatures approaching − 100 °C. Performance degradation or failure in these strategic applications can result in unpredictable and potentially hazardous scenarios [25–28]. Therefore, achieving electrochemical stability and safety under such challenging conditions is no longer optional but a mandatory objective in next-generation LIB research and development.

However, commercial LIBs, which typically consist of graphite anodes, layered oxide cathodes, and organic liquid electrolytes, suffer from severe limitations at low temperatures [29–34]. At sub-zero temperatures, the viscosity of the electrolyte rapidly increases, suppressing ionic conductivity and significantly reducing the mobility of lithium (Li)-ions [35–37]. The increased resistance at the electrode–electrolyte interface hinders charge-transfer kinetics, leading to Li plating, capacity loss, and poor reversibility [38–42]. The liquid electrolyte may become flammable or even explosive when frozen and reheated, raising serious safety concerns [14, 43–45]. These fundamental limitations show that the current configuration of LIBs is not suitable for low-temperature applications. While various additives in liquid electrolytes have been proposed, their failure to perform under extremely low-temperature conditions highlights the critical limitations of current approaches [46–49]. This ionic transport paralysis due to the frozen liquid electrolytes demonstrates the need for a paradigm shift in battery systems.

Conventional LIBs face inherent limitations at sub-zero temperatures due to their reliance on ion transport through liquid electrolytes and charge transfer across liquid–solid interfaces. Extremely low temperatures suppress mass transport and hinder the solvation/desolvation of Li-ions, which in turn impedes reaction kinetics [50, 51]. Notably, the absence of a desolvation process at the solid–solid interface removes a major source of charge-transfer resistance, enabling more efficient interfacial reactions at low temperatures [52, 53]. The solid electrolytes (SEs) exhibit lower temperature sensitivity and can maintain relatively stable ionic conductivity even at sub-zero temperatures [54–56]. These features make all-solid-state batteries (ASSBs) a strong candidate to replace conventional LIBs in demanding low-temperature environments. Although continued development is required, particularly in understanding mechanisms and performance optimization, ASSBs are considered a promising solution for overcoming the intrinsic limitations of conventional LIBs in extremely low-temperature conditions.

This review provides a comprehensive overview of recent progress in addressing low-temperature challenges, with a particular focus on ASSBs. We summarize the limitations of commercial technologies and outline research directions aimed at overcoming these barriers. In particular, we discuss key challenges in ASSBs, including SE design, interfacial engineering, and electrode optimization, together with strategies that address transport and stability issues. Progress in these technologies will not only enable next-generation batteries capable of reliable operation in polar, military, and aerospace applications but also support the broader transition toward safe and sustainable energy storage technologies.

## History

**Fig. 2:**
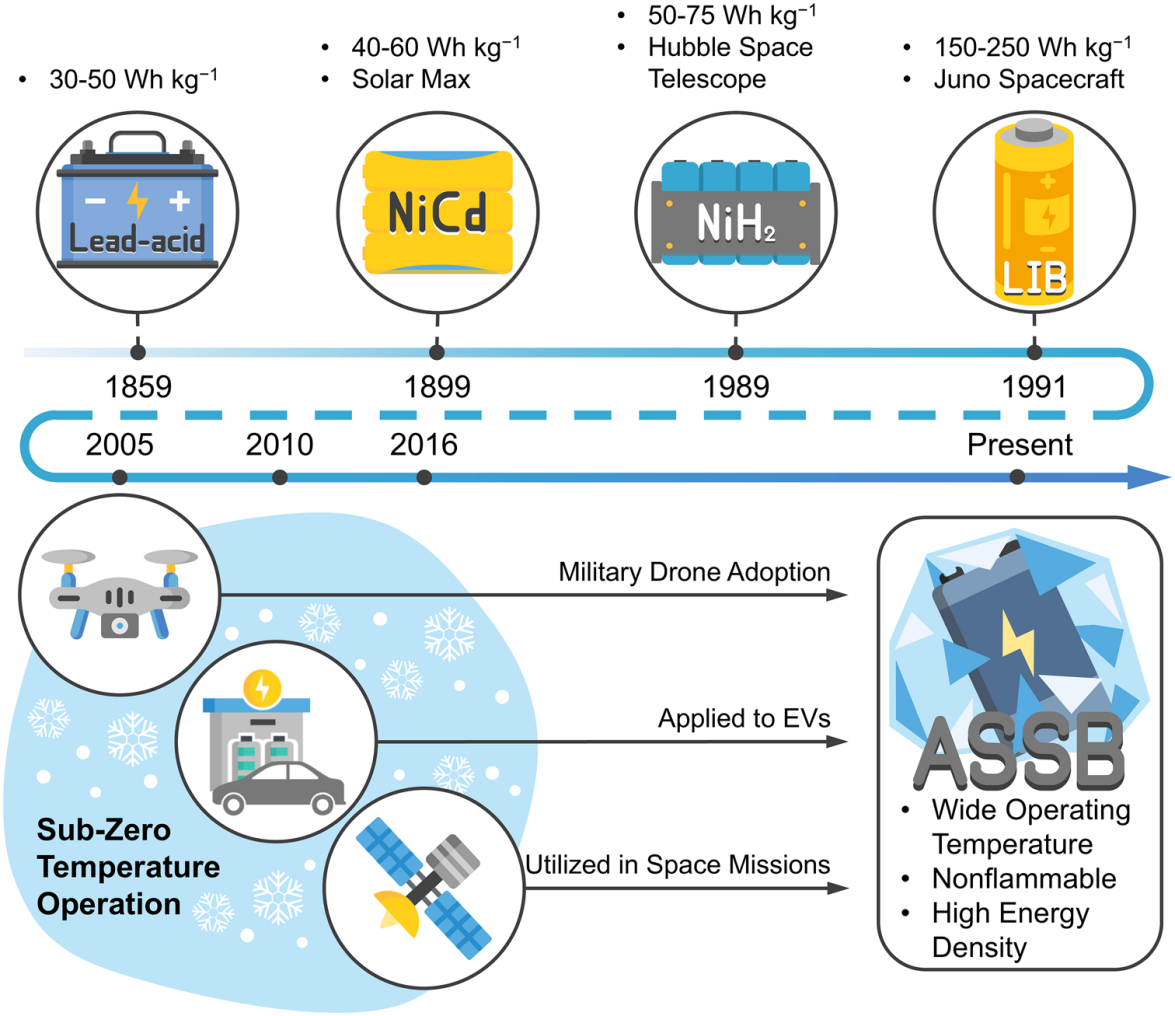
Overview of battery technology evolution (lead-acid to LIBs), highlighting the comparative Li-ion transport behavior in liquid electrolytes versus SEs under low-temperature conditions

Historically, no batteries have been specifically engineered for low-temperature environments. In practice, applications operating in extreme conditions, such as those encountered in polar expeditions, military operations, and aerospace missions, have relied on the mainstream battery technologies of their era. However, when operated under harsh sub-zero conditions, these systems often suffered from severe performance degradation and stability issues. To address these challenges, energy storage technologies have evolved over the past 150 years across various configurations and chemistries (Fig. 2). Therefore, a historical review of battery use in such environments offers valuable insights into how each system responded to these challenges and where its fundamental limitations were revealed.

The lead-acid battery, invented in 1859, featured a simple structure and low production cost and could operate at temperatures down to approximately − 20 °C [57–59]. However, its low energy density (30–50 Wh kg^−1^), heavy weight, and rapid degradation under deep discharge limited its suitability for sustained operation in extreme environments [60]. Subsequently, silver-zinc (Ag–Zn) batteries were developed for aerospace applications, offering higher energy density (70–120 Wh kg^−1^) and fast discharge capability [61, 62]. Despite their improved performance, their practical use was constrained by electrolyte freezing at low temperatures, reduced ionic conductivity, and limited cycle life [63–65]. These limitations highlighted the need for alternative battery chemistries capable of delivering reliable performance under low-temperature conditions.

Nickel–cadmium (Ni–Cd) batteries, first developed in 1899, offered an energy density of around 50–75 Wh kg^−1^ and maintained stable discharge characteristics even below − 20 °C [66–68]. These features enabled their widespread use in aircraft, communication devices, and military systems. However, Cd toxicity, memory effects, and increasing environmental regulations significantly restricted their continued use [69, 70]. Nickel–hydrogen (Ni–H_2_) batteries were subsequently introduced for applications requiring long cycle life and high stability [71]. They demonstrated reliable operation under sub-zero conditions and were adopted in spacecraft such as Intelsat V and the Hubble Space Telescope [61]. Nevertheless, bulky pressure systems, high cost, relatively low-energy density, and low packing efficiency remained major limitations [72].

A major turning point in battery technology came in 1991 with the commercialization of LIBs by Sony [73]. Owing to their high-energy density (150–250 Wh kg^−1^), long cycle life, and lightweight design, LIBs became the dominant power source for portable electronics, EVs, and even deep-space applications [74]. Before their introduction, deep-space missions in extremely cold environments relied mainly on plutonium-based radioisotope thermoelectric generators (RTGs), which provided stable long-duration power but were heavy, costly, and subject to strict safety regulations [75–77]. The development of LIBs, together with advances in solar cell technology, enabled battery-based power systems for such harsh environments [78]. However, LIB performance in extreme cold remains fundamentally limited [79]. At sub-zero temperatures, the increased viscosity of organic liquid electrolytes hinders Li-ion desolvation and slows interfacial reaction kinetics, leading to higher internal resistance, reduced output, and delayed charging. Under more severe cold conditions, these limitations become more pronounced, and Li plating during charging poses serious safety risks. These challenges, already observed in practical applications such as EV start failures and charging delays, have intensified interest in safer and more robust battery systems for severe cold environments.

Among the key performance requirements, ensuring reliability and safety under extreme conditions has become increasingly critical for next-generation battery systems. Under harsh sub-zero conditions, the physical limitations of liquid electrolytes become increasingly evident. Therefore, ASSBs that use non-flammable and non-freezing SEs offer a practical solution. Unlike organic liquid electrolytes, SEs do not freeze, suppress volumetric expansion and contraction, and allow for more stable electrode–electrolyte interfaces. The compatibility of SEs with high-capacity anode materials, such as Li metal, also offers significant potential for achieving higher energy density [80]. These advantages position ASSBs as a key technology for future energy storage systems capable of reliable operation under extremely low-temperature conditions, with applications spanning military, polar, and aerospace fields. Consequently, intensive and concerted research efforts are urgently directed toward developing materials and fabrication strategies that can guarantee superior performance and uncompromising safety in these extreme environments.

## Challenges in Low-Temperature ASSBs

Conventional LIBs face serious limitations under sub-zero conditions. At low temperatures, liquid electrolytes become highly viscous and even freeze, resulting in a sharp decrease in Li-ion conductivity and sluggish charge-transfer kinetics [81–85]. Although various electrolyte additives have been investigated to lower the freezing point and enhance ionic mobility, practical systems often rely on external heaters or thermal insulation to remain functional [86–88]. These approaches, however, provide only temporary relief and fail to address the fundamental limitations of liquid electrolytes. Overcoming these barriers calls for a transition toward SEs, which are inherently non-volatile, non-flammable, and free from freezing issues, thereby offering a strong foundation for next-generation ASSBs capable of reliable operation in harsh low-temperature environments.

**Fig. 3:**
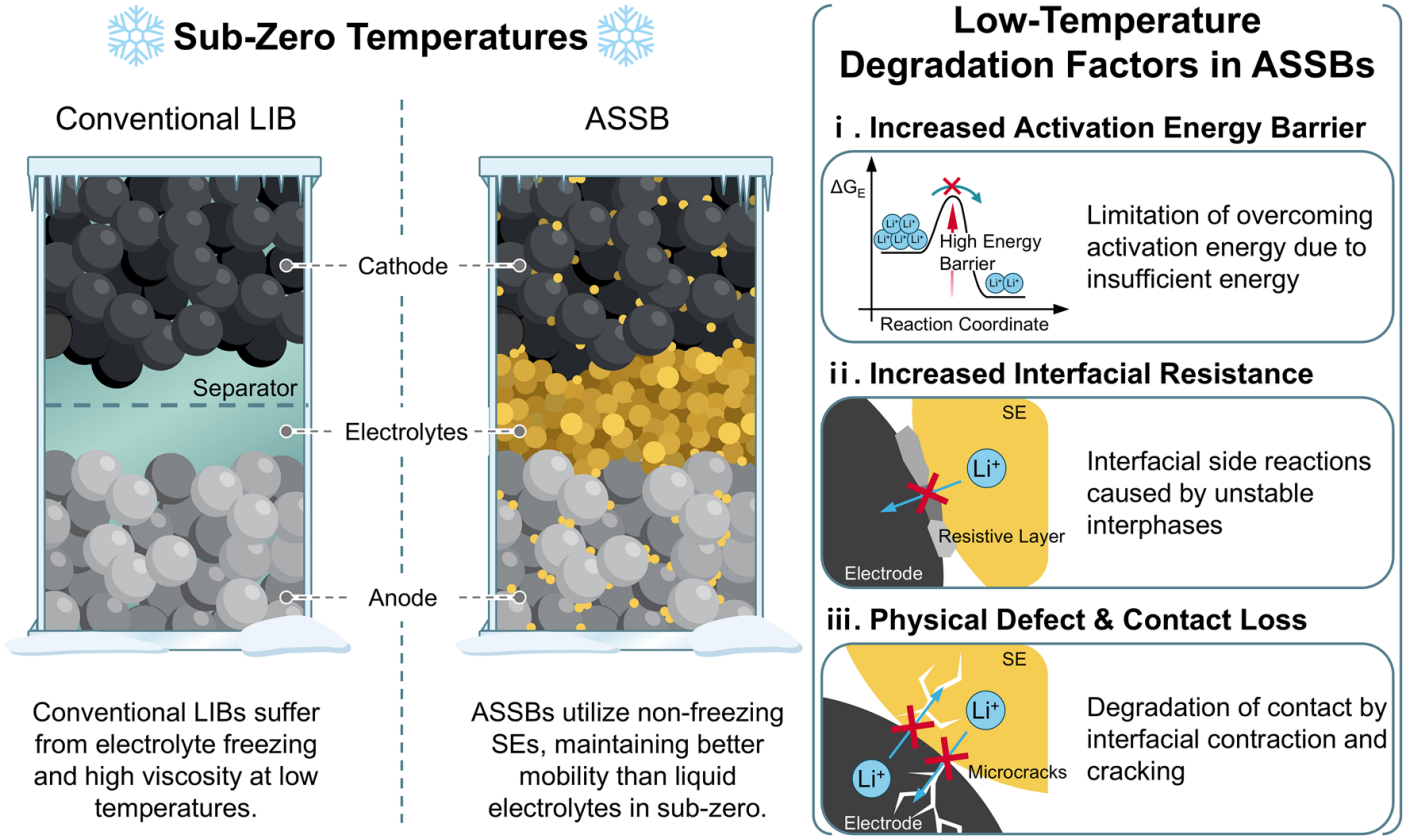
Comparison of conventional LIBs and ASSBs, and degradation factors in low-temperature ASSBs

At extremely low temperatures, the overall electrochemical reaction rate decreases, and ASSBs also experience significant performance degradation [89, 90]. As shown in Fig. 3, this degradation can be attributed to three main factors: (i) insufficient thermal energy for Li-ions to overcome activation energy barriers for solid–solid conduction, (ii) increased interfacial resistance due to side reactions, and (iii) physical issues such as microcracks and contact loss. Li-ion transport in ASSBs involves multiple steps, including diffusion within active materials, ionic conduction through the bulk SE, and charge transfer across electrode–electrolyte interfaces [91, 92]. This complexity makes it difficult to identify the exact bottlenecks responsible for performance loss at low temperatures. Recent studies have suggested that the rate-determining step in ASSBs shows temperature-dependent behavior, but a clear consensus on the fundamental mechanisms has not been reached [93, 94].

To understand the performance degradation of ASSBs at low temperatures, an integrated approach is required that considers physical, microstructural, and electrochemical factors. Li-ion transport across solid–solid interfaces follows a thermally activated hopping mechanism, in which ions must overcome an activation energy barrier to move between available lattice sites or defect pathways [52, 95]. As temperature decreases, the thermal energy available to Li-ions becomes insufficient to activate a significant fraction of these conduction pathways, leading to a sharp decline in ionic mobility. This suppression of conduction pathways disrupts the multi-step transport of Li-ion through the electrolyte network and induces significant polarization, which in turn causes localized Li-ion depletion near the active material/electrolyte interface [96]. The non-uniform Li-ion flux promotes uneven Li deposition and dendritic plating at the anode, accelerating capacity fade and posing severe safety hazards, including the risk of internal short-circuiting [97, 98].

In addition to limitations in Li-ion transport, interfacial side reactions critically affect the performance of ASSBs at low temperatures. Chemically unstable interphases form between the electrode and the SE, negatively affecting both ionic conduction and charge transfer [99]. For the cathode materials, poor interfacial compatibility with SEs, low intrinsic electronic conductivity, and restrictions on the use of conductive additives limit interfacial reactivity and stability [100–103]. At extremely low temperatures, electrochemical reactions proceed more slowly. Physical issues, such as interfacial contraction and cracking, can further deteriorate contact, leading to increased resistance and performance decline [104]. Such chemical and physical instability remains a key barrier to stable operation of ASSBs under extreme low-temperature conditions.

These interconnected issues reflect the inherent complexity of low-temperature operation in ASSBs and highlight the need for comprehensive solutions. It cannot be solved by improving a single material or component alone. Challenges such as maintaining ionic conductivity, reducing interfacial resistance, ensuring stable physical contact, and achieving chemical compatibility between electrodes and electrolytes are all closely linked. To overcome these challenges, a system-level design approach is needed that considers all components together rather than in isolation. In the following sections, we explore strategies for designing SEs and electrode materials to address these challenges in detail.

## Materials Design for Low-Temperature ASSBs

To address the challenges associated with low-temperature operation, three main strategies have been investigated in ASSB systems.

1. Improvement of charge transport: Employing SEs with low activation energy barriers and high intrinsic ionic conductivity, together with active materials exhibiting fast reaction kinetics, enhances overall charge-transfer efficiency.
2. Suppression of side reactions: Minimizing the formation of Li-ion-insulating decomposition products at electrode-SE interfaces prevents the degradation of Li-ion diffusion kinetics and the increase in charge-transfer resistance.
3. Enhancement of physical connectivity: Maximizing the solid–solid interfacial contact between electrodes and SEs promotes more efficient Li-ion exchange across interfaces

In the following sections, we review in detail the strategies developed to improve the low-temperature performance of ASSBs, focusing on advances made in SEs, cathodes, and anodes. The electrochemical performance of each strategy is summarized in Tables 1, 2 and 3.

**Table 1:** Summary of electrochemical performance of ASSBs at sub-zero temperatures enabled by charge transport improvement strategies

Fig	Cathode composition (wt%)				Anode	Solid electrolyte layer	CAM loading (mg cm^−2^)	Stack pressure (MPa)	C rate	Reversible capacity (mAh g^−1^)	Cycle retention (%)	Temp. (°C)	References
	Active material	Solid electrolyte	Binder	Additive									
4a-b	70(LiFePO_4_)	–	10(PAA)	20(Super P)	Li	BStSi	–	–	0.1	~ 55.9 (@300 cycles)	–	− 20	[80]
4c-d	80(Li_4_Ti_5_O_12_)	–	10(PVdF)	10(Super P)	Li	CPE w/ *p*-CN-SiO_2_	1.65	–	0.4	151 (@50 cycles)	–	− 10	[105]
4e-g	70(LiCoO_2_)	30(1.6Li_2_O-TaCl_5_)	–	–	Li-In	1.6Li_2_O-TaCl_5_/Li_10_GeP_2_S_12_	8.9	80	0.2	93.7 (@100 cycles)	79.2 (@300 cycles)	− 10	[106]
4h-i	70(LiCoO_2_)	25(L_1.25_NTCl)	–	5(VGCF)	Li-In	L_1.25_NTCl/LGPS	4.5	–	0.1	137.6 (@100 cycles)	83.5 (@100 cycles)	− 30	[96]
5a-b	70(LiCoO₂@LiNbO₃)	30(Li_9.54_(Si_0.6_Ge_0.4_)_1.74_P_1.44_S_11.1_Br_0.3_O_0.6_)	–	–	Li-In	Li_10.25_P_3_S_12.25_I_0.75_	171	100	0.025	101.2 (@1 cycle)	–	− 10	[108]
5c-d	70(LiNi_0.6_Mn_0.2_Co_0.2_O_2_)	30(Li_5.5_PS_4.5_Cl_1.5_)	–	–	Li-In	Li_5.5_PS_4.5_Cl_1.5_	8.9	–	0.2	81.2 (@200 cycles)	97 (@200 cycles)	− 20	[110]
5e	60(LiCoO_2_)	35(Li_3_InCl_6_)	–	5(VGCF)	Si + Li_6_PS_5_Cl + VGCF	Li₆.₈Si₀.₈As₀.₂S₅I	3.8	–	0.1	~ 53 (@1 cycle)	–	− 40	[111]
8a-b	70(LiCoO_2_)	30(Li_3_InCl_6_)	–	0.5 ~ 10(CNT)	Li-In	Li_6_PS_5_Cl	8 ~ 9	–	0.4	100.4 (@300 cycles)	89.2 (@300 cycles)	− 10	[109]
8c-d	50 ~ 60(Cu_2_S)	40 ~ 50(Li_6_PS_5_Cl)	–	–	Li-In	Li_6_PS_5_Cl	1.5 ~ 2.4	25	–	~ 400 (@200 cycles)	–	− 20	[124]
8e-f	31.25(FeS_2_)	62.5(Li_6.8_Si_0.8_As_0.2_S_5_I)	–	6.25(VGCF)	Li-In	Li_6.8_Si_0.8_As_0.2_S_5_I	2	–	0.1	226.8 (@1 cycle)	–	− 60	[94]
8g-h	42(S_7_Se_0.5_Te_0.5_)	40(Li_6_PS_5_Cl)	–	18(Ketjen black)	Li-In	Li_6_PS_5_Cl	1.34	20	0.1	559.9 (@100 cycles)	98.4 (@100 cycles)	− 20	[126]
8i-j	31.25(Mo_0.5_Ti_0.5_S_4_)	62.5(Li_6_PS_5_Cl)	–	6.25(VGCF)	Li-In	Li_6_PS_5_Cl	2	–	0.1	~ 600 (@45 cycles)	86.7 (@45 cycles)	− 20	[127]
10e-f	70(LiNi_0.8_Co_0.1_Mn_0.1_@LiNbO_3_)	30(Li_6_PS_5_Cl)	–	–	Li_0.85_Si	Li_6_PS_5_Cl	~ 13.4	–	0.05	36.4 (@1 cycle)	–	− 30	[134]

**Table 2:** Summary of electrochemical performance of ASSBs at sub-zero temperatures enabled by side reaction suppression strategies

Fig	Cathode composition (wt%)				Anode	Solid electrolyte layer	CAM loading (mg cm^−2^)	Cell press. (MPa)	C rate	Reversible capacity (mAh g^−1^)	Cycle retention (%)	Temp. (°C)	References
	Active material	Solid electrolyte	Binder	Additive									
6c-d	70(LiFePO_4_)	10(Homo-SPE)	10(PVdF)	10(Super P)	Li	S-LHCE	1 ~ 2	–	0.1	100 ~ 110 (@50 cycles)	–	− 10	[117]
6e-f	70(LiCoO_2_@LiNbO_*x*_)	30(Li_3_InCl_6_)	–	–	Li-In	Li_3_InCl_6_/Li_6_PS_5_Cl	4.5	–	0.05	99.9 (@1 cycle)	85.9 (@100 cycles)	− 10	[118]
9a-b	70(LiNi_0.7_Co_0.1_Mn_0.2_O_2_@LiNbO_3_)	30(Li_5.5_PS_4.5_Cl_1.5_)	–	–	Li-In	Li_5.5_PS_4.5_Cl_1.5_	1.8	–	0.05	79.3 (@1 cycle)	–	− 20	[128]
9c-d	50(LiNi_0.5_Co_0.2_Mn_0.3_O_2_@LiNbO_3_ v/v%)	50(Li_6_PS_5_Cl v/v%)	–	–	Li-In	Li_6_PS_5_Cl	12.5	–	0.1	~ 38 (@1 cycle)	–	− 40	[129]
9e-f	60(LiNi_0.9_Co_0.05_Mn_0.05_O_2_)	35(Li_3_InCl_6_)	–	5(VGCF)	Li-In	Li_6_PS_5_Cl	3.8	–	0.1	57.3 (@1 cycle)	–	− 30	[130]
9 g-h	70(LiNi_0.83_Co_0.11_Mn_0.06_O_2_)	30(Li-Ta-O-Cl)	–	–	Li-In	Li-Ta-O-Cl/LGPS	4.5	80	1	~ 62 (@3000 cycles)	85 (@3000 cycles)	− 10	[131]
10c-d	80(LiNi_0.8_Co_0.1_Mn_0.1_@boron-based coating)	20(Li_6_PS_5_Cl)	0.5(PTFE)	3(VGCF)	Si + PVdF	Li_6_PS_5_Cl	20	50	0.075	~ 53 (@1 cycle)	–	− 20	[135]

**Table 3:** Summary of electrochemical performance of ASSBs at sub-zero temperatures enabled by physical connectivity enhancement and multiple strategies

Fig	Cathode composition (wt%)				Anode	Solid electrolyte layer	CAM loading (mg cm^−2^)	Cell press (MPa)	C rate	Reversible capacity (mAh g^−1^)	Cycle retention (%)	Temp. (°C)	References
	Active material	Solid electrolyte	Binder	Additive									
7a-b	80(LiFePO_4_)	–	5(PVdF + EVC-LiTFSI)	15(Carbon black)	Li	In situ polymerized VEC	2.4 ~ 4.0	–	0.1	104 (@1 cycle)	–	− 15	[119]
7c-d	80(LiFePO_4_)	–	10(PVdF)	10(Super P)	Li w/ LiF-rich SEI	PEO-b-PA/LiTFSI/SN20@GF	3.5	–	0.2	73.4 (@1200 cycles)	81.5 (@1200 cycles)	− 20	[120]
7e-f	60(sulfur)	–	10(PVdF)	30(Conductive carbon)	Li	In situ polymerized DOL	0.5 ~ 1	–	–	700 (@60 cycles)	–	− 20	[121]
7g-h	60(LiFePO_4_)	30(PEO-LiClO_4_-SN)	-	10(Super P)	Li	LCPE-60	1	–	0.1	87 (@160 cycles)	–	− 10	[122]
7i-j	80(LiNi_0.8_Co_0.15_Al_0.05_O_2_)	–	10(PVdF)	10(Super P)	Li	CPCE	11	–	0.1	154.5 (@1 cycle)	~ 94 (@15 cycles)	− 20	[123]
9i-j	65(LiNi_0.9_Co_0.06_Mn_0.04_O_2_)	35(Li_3_InCl_6_)	–	–	Li	Li_6_PS_5_Cl	4.1 ~ 5.0	50	0.1	130 (@100 cycles)	–	− 20	[132]
9k-l	70(LiNi_0.7_Co_0.1_Mn_0.2_O_2_@LiNbO_3_)	30(Li_5.5_PS_4.5_Cl_1.5_)	–	–	Li-In	Li_5.5_PS_4.5_Cl_1.5_	1.8	–	0.05	75 (@35 cycles)	84.3 (@35 cycles)	− 20	[133]
10a-b	85(LiNi_0.90_Co_0.05_Mn_0.05_O_2_@Li_2_O-ZrO_2_)	15(Li_6_PS_5_Cl)	1.5(PTFE)	3(Carbon nanofiber)	Ag + carbon black + PVdF	Li_6_PS_5_Cl	–	2	0.1	~ 100 (@1 cycle)	–	− 10	[136]

### Solid Electrolyte

Conductivity of SEs is considered the most critical factor for operating low-temperature ASSBs. Although the SEs generally exhibit a wide operating temperature range, they demonstrate significantly reduced conductivity compared to operation at room temperature, which leads to severe polarization and capacity loss. The sluggish ion migration also limits charge transfer across electrode–electrolyte interfaces, increasing internal resistance and hindering Li-ion exchange while operating at sub-zero temperatures. The ionic transport properties of the SE primarily govern the overall kinetics of the battery. Therefore, many recent studies have focused on enhancing ionic conductivity. At the same time, researchers are tackling additional challenges such as oxidative decomposition and poor interfacial particle contact. This section reviews representative strategies for enhancing ionic conductivity, suppressing chemical and electrochemical decomposition, and improving interfacial contact with electrodes.

#### Enhancing Ionic Conductivity

**Fig. 4:**
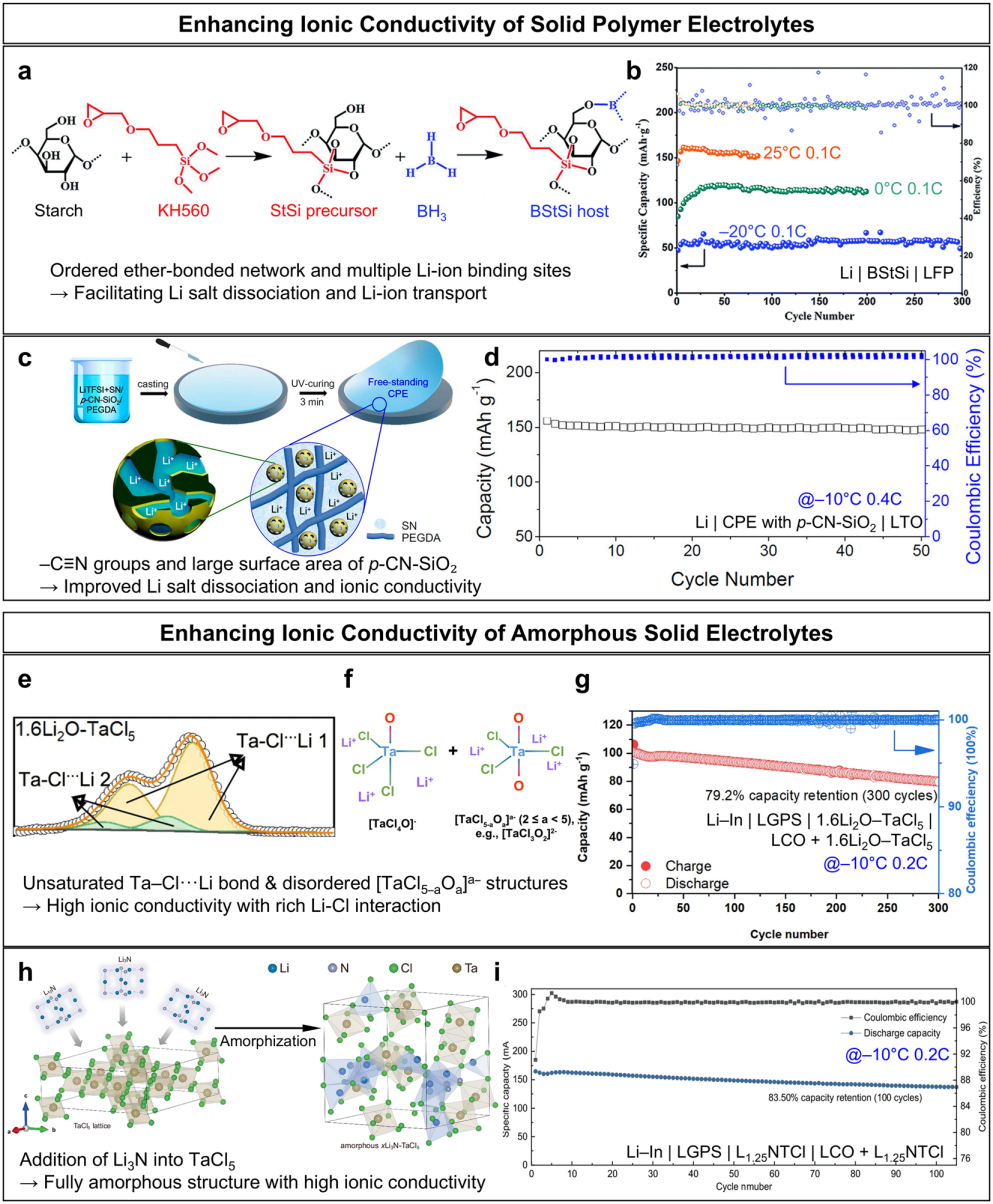
**a** Schematic of the synthesis process and proposed chemical structures of BStSi SPE (adapted from Lin et al., Copyright 2019, Royal Society of Chemistry) [80]. **b** Cycle performance of Li | BStSi | LFP cells at various temperatures (25 °C, 0 °C, – 20 °C) at 0.1C (adapted from Lin et al., Copyright 2019, Royal Society of Chemistry) [80]. **c** Schematic illustration of the fabrication process of CPE incorporating *p*-CN–SiO_2_ nanoparticles via UV curing (adapted with permission from Kwon et al., Copyright 2017, American Chemical Society) [105]. **d** Cycle performance of the Li | CPE with *p*-CN–SiO_2_ | LTO cell at –10 °C under 0.4C (adapted with permission from Kwon et al., Copyright 2017, American Chemical Society) [105]. **e** Cl 2*p* XPS for 1.6Li_2_O-TaCl_5_ amorphous SE (adapted from Zhang et al., Copyright 2023, Springer Nature) [106]. **f** Schematic of the local structures in *x*Li_2_O-TaCl_5_ amorphous SE (coordination environment and possible geometry shown; bond angles and Li-ion numbers are illustrative and not accurate) (adapted from Zhang et al., Copyright 2023, Springer Nature) [106]. **g** Cycle performance of the Li-In | LGPS | 1.6Li_2_O-TaCl_5_ | LCO + 1.6Li_2_O-TaCl_5_ cell at –10 °C under 0.2C (adapted from Zhang et al., Copyright 2023, Springer Nature) [106]. **h** Schematic illustration of Li_3_N incorporation into TaCl_5_ for amorphous *x*Li_3_N-TaCl_5_ formation (left); structure and coordination polyhedra from AIMD simulations (right) (adapted from Hong et al., Copyright 2025, Springer Nature) [96]. **i** Cycle performance of the Li-In | LGPS | L_1.25_NTCl | LCO + L_1.25_NTCl cell at –30 °C under 0.1C (adapted from Hong et al., Copyright 2025, Springer Nature) [96]

Solid polymer electrolytes (SPEs) have traditionally required high operating temperatures, often near 60 °C, due to their low ionic conductivity and low Li-ion transference numbers even at room temperature. Recent progress has been directed toward promoting Li salt dissociation and increasing ionic conductivity by tailoring chemical structures, with particular attention to performance at low temperatures. Lin et al. introduced a dual cross-linking strategy: first, the bio-based molecule starch—composed of six-membered glucose rings and alkyl chains—was cross-linked using γ-(2,3-epoxypropoxy)-propyltrimethoxy-silane (KH560), forming –O–Si–O– bonds with the –OH groups in starch (resulting in the formation of starch–Si, or StSi); second, residual –OH groups were further cross-linked using BH_3_, yielding B–starch–Si (BStSi) (Fig. 4a) [80]. This dual cross-linked BStSi provides an ordered ether-bonded network and multiple Li-ion binding sites, greatly facilitating Li salt dissociation and Li-ion transport. Owing to its relatively low interfacial resistances, the BStSi SPE exhibits exceptional ionic conductivities of 3.10 × 10^−4^ S cm^−1^ at room temperature, 1.23 × 10^−4^ S cm^−1^ at 0 °C, and 3.10 × 10^−5^ S cm^−1^ at − 20 °C. These values are several orders of magnitude higher than those of conventional poly(ethylene oxide) (PEO)-based electrolytes, which exhibit only 3.42 × 10^−8^ S cm^−1^ at 0 °C, thereby allowing efficient ion transfer even at low temperatures. When assembled in a Li | BStSi | LiFePO_4_ (LFP) cell operated at 0.1C, the cell delivers an average discharge capacity of 55.9 mAh g^−1^ over 300 cycles at − 20 °C, corresponding to 31% of the theoretical capacity of LFP, demonstrating both excellent low-temperature performance and cycling stability (Fig. 4b).

In addition to in situ structural design strategies such as dual cross-linking, ex situ incorporation of functional materials with desirable chemical structures can also be employed to chemically modify the polymer matrix and enhance ionic conductivity at sub-zero temperatures. Kwon et al. incorporated nitrile-functionalized SiO_2_ nanoparticles with mesopores (*p*-CN-SiO_2_) into a succinonitrile(SN)-based polymer electrolyte composed of poly(ethylene glycol) diacrylate (PEGDA) to enhance ionic conductivity (Fig. 4c) [105]. This UV-cured composite polymer electrolyte (CPE) formed a free-standing and flexible membrane, beneficial for practical applications. The addition of *p*-CN-SiO_2_ significantly improved the low-temperature ionic conductivity of the CPE (0.9 × 10^−4^ S cm^−1^ at − 20 °C), compared to CPEs incorporating conventional SiO_2_ nanoparticles or non-porous CN-functionalized SiO_2_. This enhancement is attributed to the improved Li salt dissociation induced by the –C≡N groups, while the mesoporous structure further amplified this effect by increasing the –C≡N surface area. As a result, a Li | CPE with *p*-CN-SiO_2_ | Li_4_Ti_5_O_12_ (LTO) cell maintained a high discharge capacity of 151 mAh g^−1^ even after 50 cycles at − 10 °C (Fig. 4d).

Amorphous SEs have attracted increasing attention for low-temperature operation due to their intrinsic softness, broad compositional tunability, and lack of grain boundaries, which are major contributors to ionic transport resistance under such conditions. However, their relatively low ionic conductivity remains a key limitation. Strategies such as tuning interatomic interactions or increasing the degree of amorphization within the SE have shown effectiveness in enhancing ionic conductivity. Zhang et al. developed a family of Li-based oxychloride amorphous SEs (*x*Li_2_O–TaCl*y*, 0.8 ≤ *x* ≤ 2, *y* = 5 or 4) [106]. At Li_2_O content above *x* = 1.6, the formation of unsaturated Ta–Cl···Li bonds was observed (Fig. 4e), which is beneficial for Li-ion migration due to the large radius, high polarizability, and weak interaction of Cl⁻ with Li⁺. Additionally, disordered structures such as [TaCl_5−a_O_a_]^a^^–^ (1 ≤ a < 5) were formed alongside [TaCl_4_O]⁻, promoting rich Li–Cl interactions (Fig. 4f). The optimized composition, 1.6Li_2_O–TaCl_5_, exhibited a low activation energy of 0.274 eV and a high ionic conductivity of 6.6 × 10^−3^ S cm^−1^ at 25 °C. When evaluated in a Li–In | Li_10_GeP_2_S_12_ (LGPS) | 1.6Li_2_O–TaCl_5_ | LiCoO_2_ (LCO) + 1.6Li_2_O–TaCl_5_ cell at a low temperature of – 10 °C, the cell delivered excellent electrochemical performance, showing a discharge capacity of 93.7 mAh g^−1^ after 100 cycles, and achieving a capacity retention of 79.2% after 300 cycles with an average Coulombic efficiency of 99.94% (Fig. 4g).

More recently, Hong et al. presented a new category of amorphous SEs: *x*Li_3_N–TaCl_5_ (1 ≤ 3*x* ≤ 2). TaCl_5_, known for its high amorphization tendency when mixed with other atoms, serves as the structural framework [96]. Li_3_N, a rich source of charge carriers that contribute to ionic conductivity, was incorporated at an optimal ratio (Fig. 4h). The resulting composition with 3*x* = 1.25 (denoted L_1.25_NTCl) exhibited a fully amorphous structure and the highest room-temperature ionic conductivity (5.91 mS cm^−1^) among all tested compositions. Furthermore, it demonstrated a low activation energy of 0.279 eV and maintained high ionic conductivity even at sub-zero temperatures—0.5, 0.29, and 0.07 mS cm^−1^ at − 30, − 40, and − 60 °C, respectively. At − 30 °C, a Li–In | LGPS | L_1.25_NTCl | LCO + L_1.25_NTCl cell exhibited excellent electrochemical performance, retaining 83.5% of its initial capacity after 100 cycles with a discharge capacity of 137.6 mAh g^−1^ (Fig. 4i). Note that LGPS was introduced as an interlayer above the Li–In anode to ensure interface stability.

**Fig. 5:**
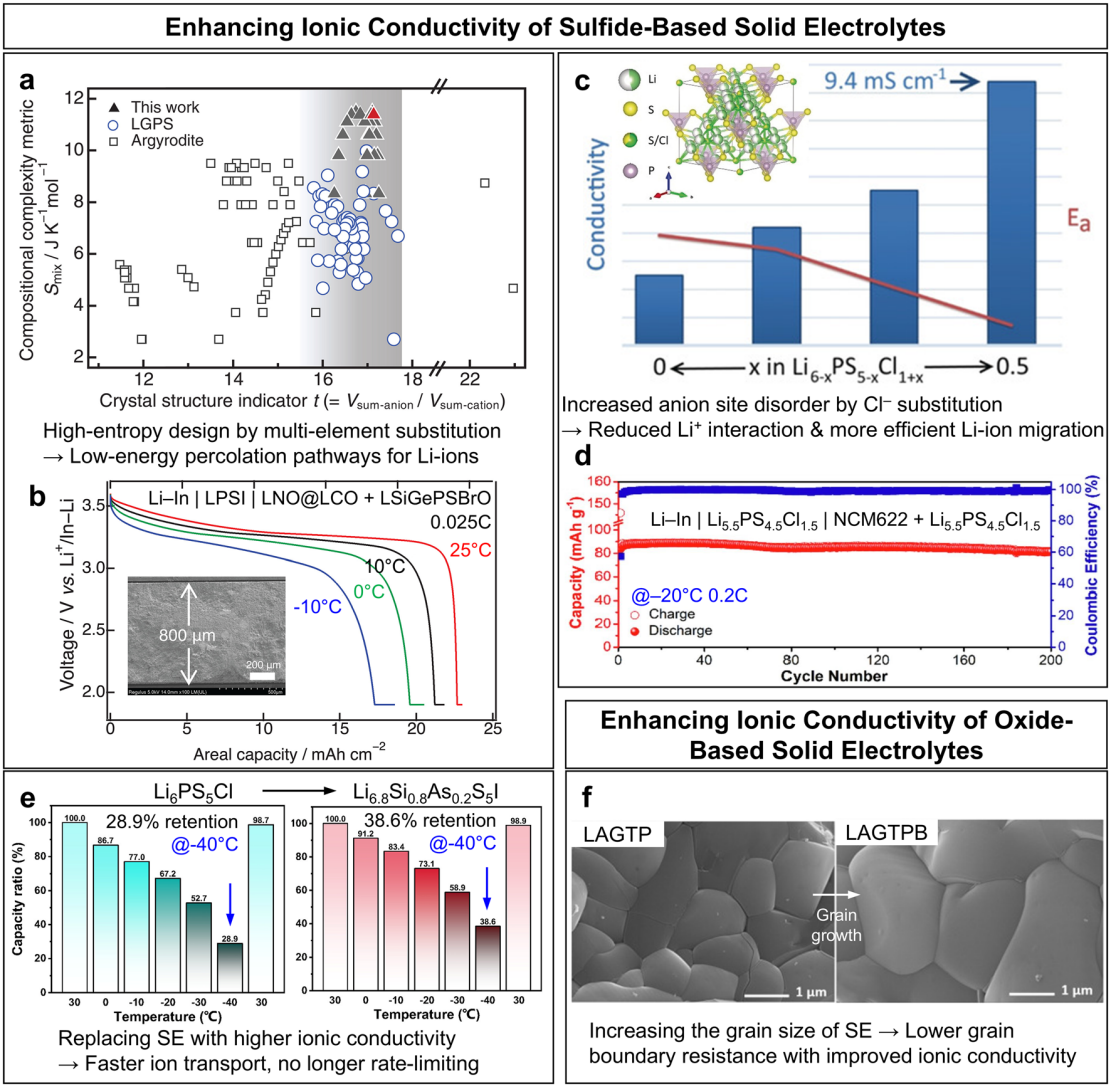
**a** Relationship between the crystal structure indicator *t* (anion-to-cation volume ratio) and the compositional complexity metric (S_mix_) for LGPS-type (open circles) and argyrodite-type (open squares) SEs. (adapted with permission from Li et al., Copyright 2023, The American Association for the Advancement of Science) [108]. **b** Discharge curves of the Li-In | LPSI | LNO@LCO + LSiGePSBrO cell at various temperatures (25 °C, 10 °C, 0 °C, – 10 °C) at 0.025C (adapted with permission from Li et al., Copyright 2023, The American Association for the Advancement of Science) [108]. **c** Schematic of Li-ion conductivities and activation energies in Li_6-*x*_PS_5-*x*_Cl_1+*x*_ (0 ≤ *x* ≤ 0.5) SEs (adapted with permission from Adeli et al., Copyright 2019, Wiley). **d** Cycle performance of the Li-In | Li_5.5_PS_4.5_Cl_1.5_ | NCM622 + Li_5.5_PS_4.5_Cl_1.5_ cell at – 20 °C under 0.2C (adapted with permission from Peng et al., Copyright 2021, Elsevier) [110]. **e** Capacity ratio of Si + LPSCl | LPSCl | LCO + LIC and Si + LPSCl | LASI-80Si | LCO + LIC cells at each temperature to that of 30 °C (adapted with permission from Lu et al., Copyright 2024, Elsevier) [111]. **f** SEM images of the fracture surfaces of LAGTP and LAGTPB (adapted with permission from Saffirio et al., Copyright 2021, Elsevier) [113]

Another promising approach to improve ionic conductivity at low temperatures is the high-entropy design strategy. It reduces potential barriers for Li-ion migration in SEs by overlapping the site energy distribution, thereby enabling low-energy percolation pathways for Li-ions [107]. Li et al. [108] developed a high-entropy sulfide-based SE by applying multi-element substitution to LGPS, a material known for its high ionic conductivity (~ 12 mS cm^−1^ at room temperature), comparable to that of liquid electrolytes. The resulting compound, Li_9.54_(Si_0.6_Ge_0.4_)_1.74_P_1.44_S_11.1_Br_0.3_O_0.6_ (LSiGePSBrO), was engineered to maximize the compositional complexity metric derived from the configurational entropy of mixing, a defining feature of high-entropy materials (Fig. 5a). Thus, LSiGePSBrO exhibited a lower activation energy and achieved a high ionic conductivity of 9 mS cm^−1^ at – 10 °C, which is comparable to that of LGPS at room temperature. Utilizing this material, a Li–In | Li_10.25_P_3_S_12.25_I_0.75_ (LPSI) | LiNbO_3_-coated LCO (LNO@LCO) + LSiGePSBrO cell delivered an exceptionally high discharge capacity of 17.3 mAh cm^−2^ (101.2 mAh g^−1^) at − 10 °C, corresponding to 75% of the capacity at 25 °C (Fig. 5b).

Argyrodite-type sulfide-based SEs have recently garnered significant attention as one of the most commercially viable candidates due to their ductility and high ionic conductivity (above 10^−3^ S cm^−1^ at room temperature). Unlike some other sulfide-based SEs, they do not contain expensive elements such as Ge, and are composed of earth-abundant, low-cost materials. Moreover, they can form a stable passivation layer with Li metal, further enhancing their compatibility for ASSB systems. Tailoring their composition has proved effective in achieving both high conductivity and favorable low-temperature performance. Adeli et al. reported a series of Li-deficient Li_6-*x*_PS_5-*x*_Cl_1+*x*_ compositions (*x* = 0–0.5), where the *x* = 0.5 composition exhibited the highest ionic conductivity of 9.4 mS cm^−1^ and the lowest activation energy of 0.29 eV at 25 °C (Fig. 5c). This improvement was attributed to enhanced anion site disorder between S^2−^ and Cl^−^ and a reduced strength of interactions between Li-ions and the surrounding anionic framework, facilitating more efficient Li-ion migration [109]. Building on this, Peng et al. synthesized Li_5.5_PS_4.5_Cl_1.5_ with a high ionic conductivity of 9.03 mS cm^−1^ on a > 10 g batch scale [110]. When assembled in a Li–In | Li_5.5_PS_4.5_Cl_1.5_ | LiNi_0.6_Mn_0.2_Co_0.2_O_2_ (NCM622) cell and tested at − 20 °C, the cell delivered a discharge capacity of 81.2 mAh g^−1^ after 200 cycles with a capacity retention of 97.0%, demonstrating excellent electrochemical performance under low-temperature conditions (Fig. 5d).

Simply increasing the ionic conductivity of the SE layer can accelerate ion conduction within the SE and significantly enhance capacity retention at low temperatures. To enhance low-temperature performance, Lu et al. substituted Li_6_PS_5_Cl (LPSCl, 3.8 mS cm^−1^ at 25 °C, activation energy of 0.35 eV) with Li_6.8_Si_0.8_As_0.2_S_5_I (LASI-80Si), a SE exhibiting significantly higher ionic conductivity (10.4 mS cm^−1^ at 25 °C) and a lower activation energy (0.20 eV) [111]. This replacement effectively accelerated ion conduction, leading to improved capacity retention of a μm-scale Si | LASI-80Si | LCO + Li_3_InCl_6_ (LIC) cell at – 40 °C with an increase from 28.9 to 38.6% relative to its capacity at 30 °C (Fig. 5e). This improvement can be attributed to a shift in the rate-limiting process of the cell. In the case of the cell with LPSCl SE layer at sub-zero temperatures, the dominant rate-limiting step was ion transport across the SE grain boundaries, with activation energies of 38.21 kJ mol^−1^ for *R*_ohm_ (primarily from grain boundary resistance) and 35.22 kJ mol^−1^ for *R*_cc_ (mainly from contact resistance at cathode particles or the current collector). However, after introducing LASI-80Si SE layer, the rate-limiting step shifted from *R*_ohm_ to *R*_cc_, with activation energies of 34.39 kJ mol^−1^ and 38.94 kJ mol^−1^, respectively, which contributed to the improved performance at low temperatures.

To enhance the low-temperature ionic conductivity of SEs, it is also important to increase the grain size and promote densification. Suitable sintering aids can be introduced to enhance densification and reduce the resistance associated with ion transport across grain boundaries [112, 113]. Saffirio et al. synthesized a NASICON-type SE, Li_1.4_Al_0.4_Ge_0.4_Ti_1.4_(PO_4_)_3_ (LAGTP) and introduced 0.05 wt% of B_2_O_3_, which is an additive known to promote the liquefaction of grain boundaries due to its relatively low melting point of 450 °C, during the melt-casting process to form B_2_O_3_-doped LAGTP (LAGTPB). The addition of B_2_O_3_ facilitated grain growth (increasing the average grain size from 1 to 2 μm) and enhanced intergranular cohesion (Fig. 5f). As a result, the grain boundary resistance was reduced, leading to improved ionic conductivity and a lowered activation energy: from 0.30 eV and 4.8 × 10^−5^ S cm^−1^ (at – 10 °C) for pristine LAGTP to 0.28 eV and 1.01 × 10^−4^ S cm^−1^ for LAGTPB.

#### Suppressing Chemical/Electrochemical Decomposition

**Fig. 6:**
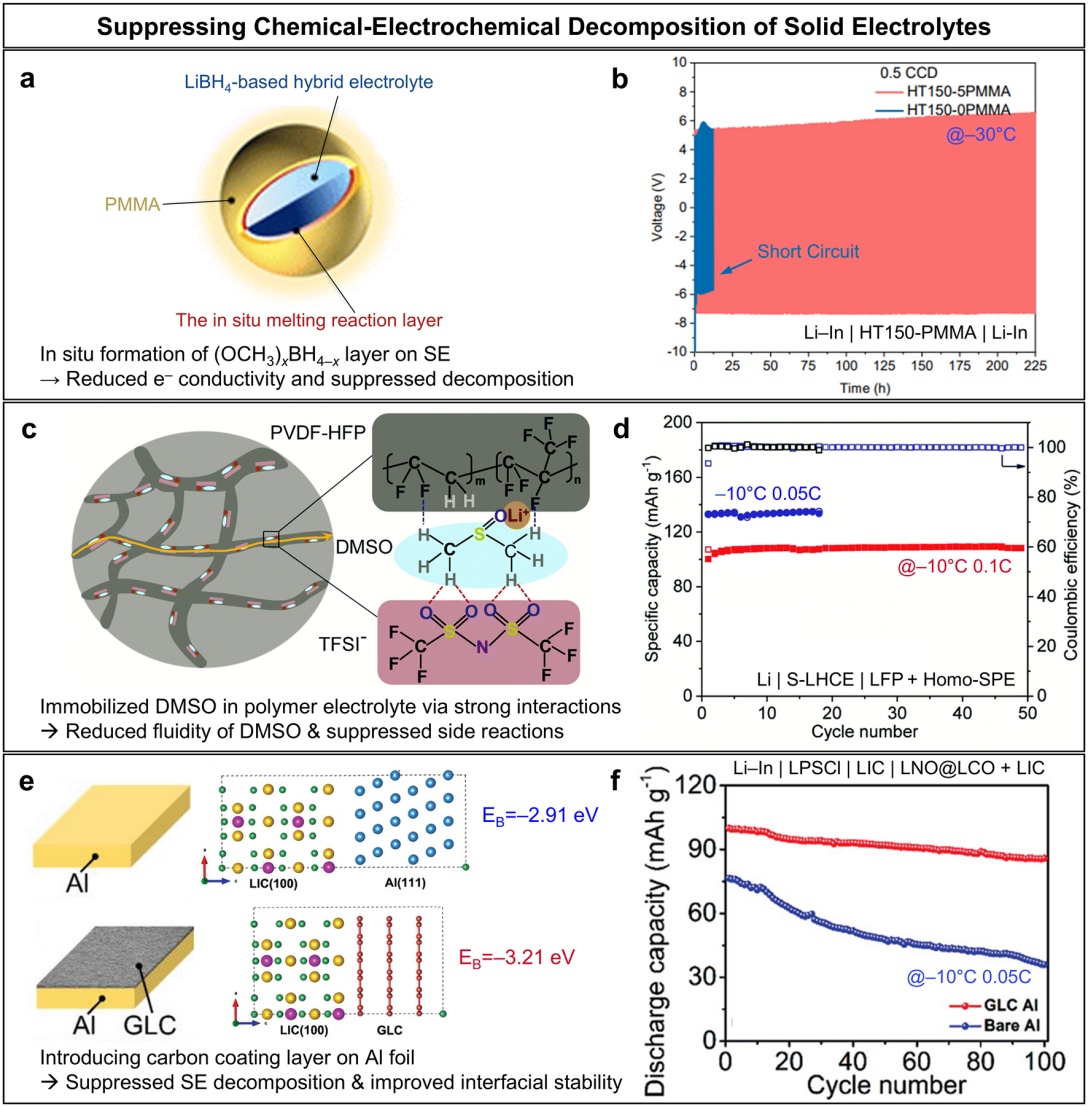
**a** Schematic illustration of in situ formation of (OCH_3_)_*x*_BH_*4−x*_ interfacial layer on LiBH_4_-based hybrid electrolyte (adapted with permission from Wei et al., Copyright 2023, Royal Society of Chemistry) [116]. **b** Li plating and stripping cycles of the Li-In | HT150 − *x*PMMA (*x* = 0.5) | Li-In cells at – 30 °C under 0.525 mA cm^−2^ (adapted with permission from Wei et al., Copyright 2023, Royal Society of Chemistry) [116]. **c** Schematic illustration of Li-ion transport model in the S-LHCE (adapted with permission from Xu et al., Copyright 2022, Royal Society of Chemistry) [117]. **d** Cycle performance of the Li | S-LHCE | LFP + Homo-SPE cell at – 10 °C under 0.05C and 0.1C (adapted with permission from Xu et al., Copyright 2022, Royal Society of Chemistry) [117]. **e** Schematic of bare and GLC-coated Al foil with LIC(100)/Al(111) and LIC(100)/GLC binding energies (adapted with permission from Deng et al., Copyright 2022, Wiley) [118]. **f** Cycle performance of the Li-In | LPSCl | LIC | LNO@LCO + LIC cells with bare Al foil and GLC-coated Al foil at – 10 °C under 0.05C (adapted with permission from Deng et al., Copyright 2022, Wiley) [118]

The chemical/electrochemical decomposition on the surface of SEs causes resistive passivation by their by-products. And thus, reducing it can serve as an effective strategy to suppress interfacial side reactions [114, 115]. Wei et al. formed a covalently bonded reaction layer ((OCH_3_)_*x*_BH_4-*x*_) on the surface of LiBH_4_ SE particles through an in situ melting reaction with polymethyl methacrylate (PMMA) (Fig. 6a) [116]. The (OCH_3_)_*x*_BH_4-*x*_ layer can effectively suppress electron exchange during the oxidation process of BH_4_^−^ due to its strong electronic localization. The in situ melting reaction was conducted at 150 °C for 1 h, and the resulting samples were labeled as HT150-*x*PMMA, where *x* represents the PMMA content in wt% (*x* = 0 or 5). Both HT150-0PMMA and HT150-5PMMA exhibit comparable Li-ion conductivities (5 × 10^−4^ S cm^−1^ at room temperature) and activation energies (0.48 eV), suggesting that the in situ melting reaction does not impede ionic transport. However, the PMMA-added sample shows a marked reduction in electronic conductivity (1.27 × 10^−7^ S cm^−1^ for HT150-0PMMA and 5.35 × 10^−10^ S cm^−1^ for HT150-5PMMA at room temperature), which effectively minimizes interfacial side reactions. It indicates a reduction by approximately three orders of magnitude upon PMMA addition. As a result, cyclic voltammetry (CV) analysis showed that HT150-5PMMA exhibits high oxidative stability with no observable oxidation peaks within the 0–10 V range. Furthermore, in Li plating and stripping tests using Li-In symmetric cells at − 30 °C, the HT150-5PMMA cell demonstrated superior cycling stability compared to the HT150-0PMMA cell, operating without short-circuiting for 225 h (Fig. 6b).

Controlling intermolecular interactions within the polymer electrolyte has been shown to suppress side reactions at the polymer electrolyte/electrode interface. Xu et al. [117] prepared a solidified localized high-concentration electrolyte (S-LHCE) using a freeze-drying method, which enhanced interfacial stability by decoupling ion pairing from ion conduction (Fig. 6c). The S-LHCE utilizes a non-solvating poly(vinylidene fluoride-*co*-hexafluoropropylene) (PVDF-HFP) polymer framework to confine the unstable solvent, dimethyl sulfoxide (DMSO), which possesses a high dielectric constant (ε_r_ ≈ 47.2) and strong Li salt solvation ability. At ultrahigh concentrations, DMSO is immobilized through strong interactions with both the PVDF-HFP matrix and TFSI⁻ anions. This molecular bridging effect not only reduces the fluidity of DMSO but also effectively suppresses side reactions with the electrode. As a result, no decomposition involving C–S_*x*_ bonding, which is associated with DMSO decomposition, was observed, as confirmed by C 1*s* X-ray photoelectron spectroscopy (XPS) analysis after etching. Consequently, electrochemical evaluation showed that the Li | S-LHCE | LFP cell delivered a stable discharge capacity of approximately 100–110 mAh g^−1^ over 50 cycles at − 10 °C (Fig. 6d).

The cathode current collector can also undergo side reactions with the SE, making its stabilization an important area of research. Deng et al. addressed this issue by introducing a graphene-like carbon (GLC) coating on the surface of an Al foil current collector to suppress interfacial reactions with the LIC SE (Fig. 6e) [118]. DFT calculations revealed that while the binding energy between Al(111) and LIC(100) was − 2.91 eV, the binding energy between GLC and LIC(100) was more favorable at − 3.21 eV, indicating a more stable interface formed between GLC and LIC(100). Electrochemical testing of Li–In | LPSCl | LIC | LNO@LCO + LIC cells showed that the cell with the GLC-coated Al foil delivered an initial discharge capacity of 99.9 mAh g^−1^ at − 10 °C, significantly higher than the 76.5 mAh g^−1^ obtained with the bare Al foil. Moreover, the capacity retention after 100 cycles was markedly improved, reaching 85.9% compared to only 47.1% for the bare Al foil (Fig. 6f). XPS analysis after cycling further confirmed that the GLC coating effectively suppressed the formation of InCl_3_, a decomposition product of LIC, indicating improved interfacial stability.

#### Improving Interfacial Contact with Electrodes

**Fig. 7:**
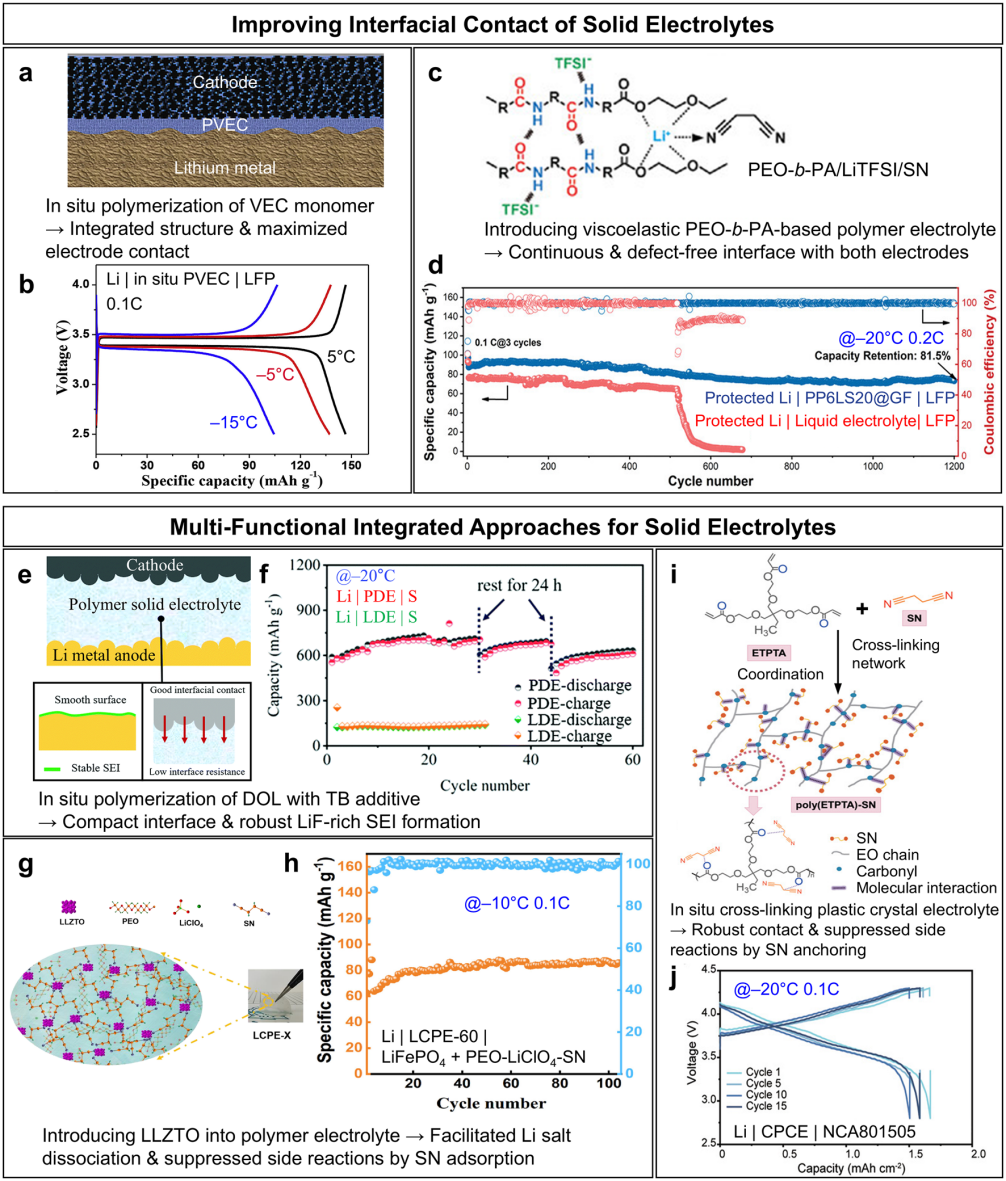
**a** Schematic illustration of the Li | in situ PVEC | Cathode cell (adapted with permission from Lin et al., Copyright 2020, Elsevier) [119]. **b** Charge–discharge curves of the Li | in situ PVEC | LFP cell cells at various temperatures (5 °C, − 5 °C, − 15 °C) at 0.1C (adapted with permission from Lin et al., Copyright 2020, Elsevier) [119]. **c** Schematic representation of PEO-*b*-PA/LiTFSI/SN chemical structure (adapted with permission from Huang et al., Copyright 2023, Wiley) [120]. **d** Cycle performance of the Li | Liquid electrolyte | LFP and Li | PP6LS20@GF | LFP cells at – 20 °C under 0.2C (adapted with permission from Huang et al., Copyright 2023, Wiley) [120]. **e** Schematic illustration of the Li | PDE | Cathode cell (adapted with permission from Xiang et al., Copyright 2022, Royal Society of Chemistry) [121]. **f** Cycle performance of the Li | LDE | S and Li | PDE | S cells at – 20 °C (adapted with permission from Xiang et al., Copyright 2022, Royal Society of Chemistry) [121]. **g** Schematic illustration of the LCPE hybrid electrolyte (adapted with permission from Zhang et al., Copyright 2023, Elsevier) [122]. **h** Cycle performance of the Li | LCPE-60 | LFP + PEO-LiClO_4_-SN cell at – 10 °C under 0.1C (adapted with permission from Zhang et al., Copyright 2023, Elsevier) [122]. **i** Schematic illustration of the chemical structures of ETPTA and SN forming a cross-linking network (left) and their molecular interaction (right), adapted with permission from Wang et al., Copyright 2022, Wiley) [123]. **j** Charge–discharge curves of Li|CPCE|NCA full cells with high cathode active material loading under − 20 °C, adapted with permission from Wang et al., Copyright 2022, Wiley) [109]

The formation of polymer electrolytes via in situ polymerization within the battery ensures the development of an integrated structure with electrodes, thereby enabling intimate interfacial contact. Lin et al. demonstrated excellent interfacial compatibility within the cell through in situ polymerization of the vinyl ethylene carbonate (VEC) liquid monomer (Fig. 7a) [119]. This approach resulted in a highly integrated electrode structure with a maximized contact area, due to excellent adhesion between layers. In contrast, the ex situ polymerization method led to noticeable interlayer gaps. Furthermore, in Li symmetric cell tests, the in situ polymerized cell exhibited significantly lower voltage polarization compared to the ex situ polymerized counterpart. The Li | in situ PVEC | LFP cell delivered a high discharge capacity of approximately 104 mAh g^−1^ at − 15 °C (Fig. 7b).

By tuning the physical properties of the polymer electrolyte, an integrated interface with both the anode and cathode can be formed. Huang et al. designed a polyether-*b*-amine (PEO-*b*-PA)-based composite polymer electrolyte via a solvent-free method, where the dynamic PA backbone could facilitate the formation of a conformal electrode–electrolyte interface (Fig. 7c) [120]. The PEO-*b*-PA/LiTFSI/SN@glass fiber (PPLS@GF) electrolyte showed similar values of storage modulus (G′) and loss modulus (G″) at − 20 °C, indicating a semisolid, gel-like state under low-temperature conditions. This viscoelastic property enabled the polymer electrolyte to penetrate the electrode structure and form physical entanglement. Moreover, the electrolyte exhibits low interfacial charge-transfer resistance even at − 20 °C. The addition of SN weakens the interaction between the polymer and Li-ions. This promotes stable Li-ion transport at the electrode and enhances ionic conductivity. Owing to these favorable interfacial characteristics, the Li@LiF-rich SEI | PPLS@GF | LFP cell demonstrated excellent electrochemical performance, delivering 73.4 mAh g^−1^ and 81.5% capacity retention after 1200 cycles at − 20 °C (Fig. 7d).

#### Multi-Functional Integrated Approaches

While the previous sections separately discussed strategies for suppressing side reactions, enhancing ionic conductivity, and improving interfacial contact, many recent studies aim to address multiple challenges simultaneously. This integrative approach reflects the practical need to improve overall cell performance under low-temperature conditions, where both interfacial instability and sluggish Li-ion transport coexist. This section highlights representative strategies that concurrently tackle these key issues, including: (i) suppressing side reactions while increasing contact area, (ii) improving ionic conductivity while mitigating side reactions, and (iii) simultaneously enhancing ionic conductivity, improving interfacial contact, and suppressing side reactions at low temperatures.

Recently, Xiang et al. demonstrated that the in situ polymerized 1,3-dioxolane (DOL) electrolyte (referred to as PDE) allowed the precursor to fully infiltrate the pores of the electrodes, followed by solidification into a compact interface (Fig. 7e) [121]. This process was facilitated by a multi-functional additive, tris(pentafluorophenyl)borane (TB), which not only initiated the polymerization but also underwent reduction at the Li surface to form a LiF-rich SEI. The decomposition of TB generates B_*x*_O_*y*_ species with O–B–O bonds that form a cross-linked covalent network, thereby contributing to a robust SEI and stabilizing the Li anode. Due to these characteristics, Li | PDE | S cell delivered a capacity of ~ 700 mAh g^−1^ for 60 cycles at − 20 °C (Fig. 7f). In contrast, a cell with liquid DOL electrolytes (LDE) exhibited negligible capacity under the same conditions, due to severe aggregation of lithium polysulfides at low temperatures, even though LDE (6.91 mS cm^−1^) showed higher ionic conductivity than PDE (1.16 mS cm^−1^) at 30 °C.

To enhance the ionic conductivity of SPEs, SN is commonly used as a plasticizer. SN facilitates Li salt dissociation and reduces the crystallinity of the polymer matrix, thereby improving ion transport. However, due to its high polarity, SN tends to react with the Li metal anode, forming undesirable by-products such as Li_3_N. To address the aforementioned concern, Zhang et al. demonstrated that incorporating Li_6.4_La_3_Zr_1.4_Ta_0.6_O_12_ (LLZTO) into a PEO-based SPE could suppress these side reactions while maintaining high ionic conductivity (Fig. 7g) [122]. DFT calculations showed that the adsorption energy of SN was stronger on the surface La^3+^ cations in LLZTO (− 3.84 eV) than on Li metal (− 2.68 eV), indicating that the N atom in SN binds more strongly to La^3+^ sites than to Li. The formation of Li_3_N after cycling was significantly suppressed when LLZTO was incorporated, as confirmed by XPS N 1*s* spectra analysis. This suppression allowed the use of a high-SN-content (60 wt%) LLZTO-containing composite polymer electrolyte (LCPE-60) in ASSBs. Owing to the suppressed side reactions with Li and a high ionic conductivity of 7.66 × 10^−4^ S cm^−1^ at room temperature, a Li | LCPE-60 | LFP + PEO-LiClO_4_-SN cell demonstrated excellent low-temperature performance, delivering 87 mAh g^−1^ after 160 cycles at − 10 °C (Fig. 7h).

Wang et al. developed an in situ cross-linked plastic crystal electrolyte (CPCE) based on SN–LiTFSI–LiDFOB to simultaneously address several challenges related to SN: the side reactions between SN and Li metal, the reduced ionic conductivity due to increased SN crystallinity at low temperatures, and the insufficient interfacial contact commonly observed in solvent-free plastic crystal electrolytes (PCEs) [123]. The employed polymer monomer, ethoxylated trimethylolpropane triacrylate (ETPTA), contains highly polar groups, which enable strong anchoring of a large amount of SN (Fig. 7i). The anchoring design significantly enhances interfacial stability with Li metal. The binding energy between ETPTA and SN was calculated to be − 0.47 eV, which is more stable than the SN–SN interaction (– 0.35 eV), indicating that SN molecules in CPCE preferentially interact with ETPTA rather than with each other. This anchoring effect effectively suppresses SN crystallization at low temperatures. Additionally, the uniform distribution of the S element from TFSI⁻ across both the electrolyte and cathode, as confirmed by scanning electron microscopy–energy-dispersive X-ray spectroscopy (SEM–EDS) mapping analysis, evidences the formation of a robust interfacial contact. As a result, a Li | CPCE | LiNi_0.8_Co_0.15_Al_0.05_O_2_ (NCA801505) cell delivered an initial discharge capacity of 1.7 mAh cm^−2^ (corresponding to 154.5 mAh g^−1^) at − 20 °C and maintained approximately 1.6 mAh cm^−2^ after 15 cycles (Fig. 7j).

#### Limitations and Trade-Offs in Solid Electrolytes

The strategies discussed above to improve the low-temperature performance of SEs, including sulfide-based systems, defect-engineered structures, and polymer/composite electrolytes, introduce inherent trade-offs associated with ion transport, mechanical integrity, and interfacial stability. Sulfide-based and defect-engineered electrolytes can deliver high ionic conductivity at low temperatures. However, their relatively low mechanical strength facilitates interfacial deformation and Li dendrite penetration. In contrast, oxide electrolytes offer superior mechanical robustness, but their higher activation energy limits Li-ion transport under sub-zero conditions, resulting in increased polarization. Polymer and composite electrolytes, which are often introduced to enhance interfacial contact and Li-ion mobility, illustrate another trade-off. While they improve interfacial compatibility and reduce contact resistance, their lower modulus and increased inactive volume fraction can compromise both mechanical stability and volumetric energy density.

Modifications intended to enhance ionic transport, such as compositional tuning and defect engineering, can also influence interfacial reactivity. Increased defect concentrations or chemically active components facilitate Li-ion migration, but can simultaneously introduce reactive sites and thermodynamic instability, promoting side reactions and unstable interphase formation. These effects become more pronounced at low temperatures, where sluggish Li-ion transport amplifies interfacial resistance growth. The use of multi-component systems and composite architectures also increases material and processing complexity. Approaches such as high-entropy compositions or hybrid electrolyte designs can improve performance but require more controlled synthesis and processing conditions, which can limit reproducibility and scalability.

These findings demonstrate that improvements in ionic conductivity, interfacial contact, and mechanical properties are strongly coupled, and enhancing one aspect often leads to compromises in others. As a result, optimizing a single property does not necessarily translate to improved overall cell performance under low-temperature conditions.

### Cathode

At sub-zero temperatures, the cathode plays a critical role in determining the overall performance of ASSBs, as sluggish Li-ion transport and marked interfacial polarization severely hinder reversible electrochemical reactions. Composite cathodes consist of CAMs, SEs, and conductive additives to ensure ionic and electronic conductivity. Electronic conductivity is generally less temperature-sensitive than ionic conductivity. Thus, poor ionic conducting behavior by solid–solid contact and progressive interfacial degradation during cycling are crucial issues. These effects are exacerbated at low temperatures, where ion migration and interfacial charge transfer are kinetically constrained. These limitations lead to increased charge-transfer resistance, reduced active material utilization, and significant capacity fading.

To mitigate these limitations, advanced cathode composites have been developed to promote efficient and stable Li-ion transport across CAM–SE interfaces. Recent advances have mainly focused on three major strategies to improve cathode performance under low-temperature conditions. These include enhancing Li-ion transport kinetics through compositional and structural engineering of CAMs and SEs, suppressing interfacial degradation by introducing protective interlayers, and constructing intimate interfacial contact through microstructural optimization and mechanical processing. The following sections summarize representative approaches based on these strategies and highlight key findings that have contributed to improving the low-temperature performance of ASSB cathodes.

#### Enhancing Li-Ion Transport Kinetics

**Fig. 8:**
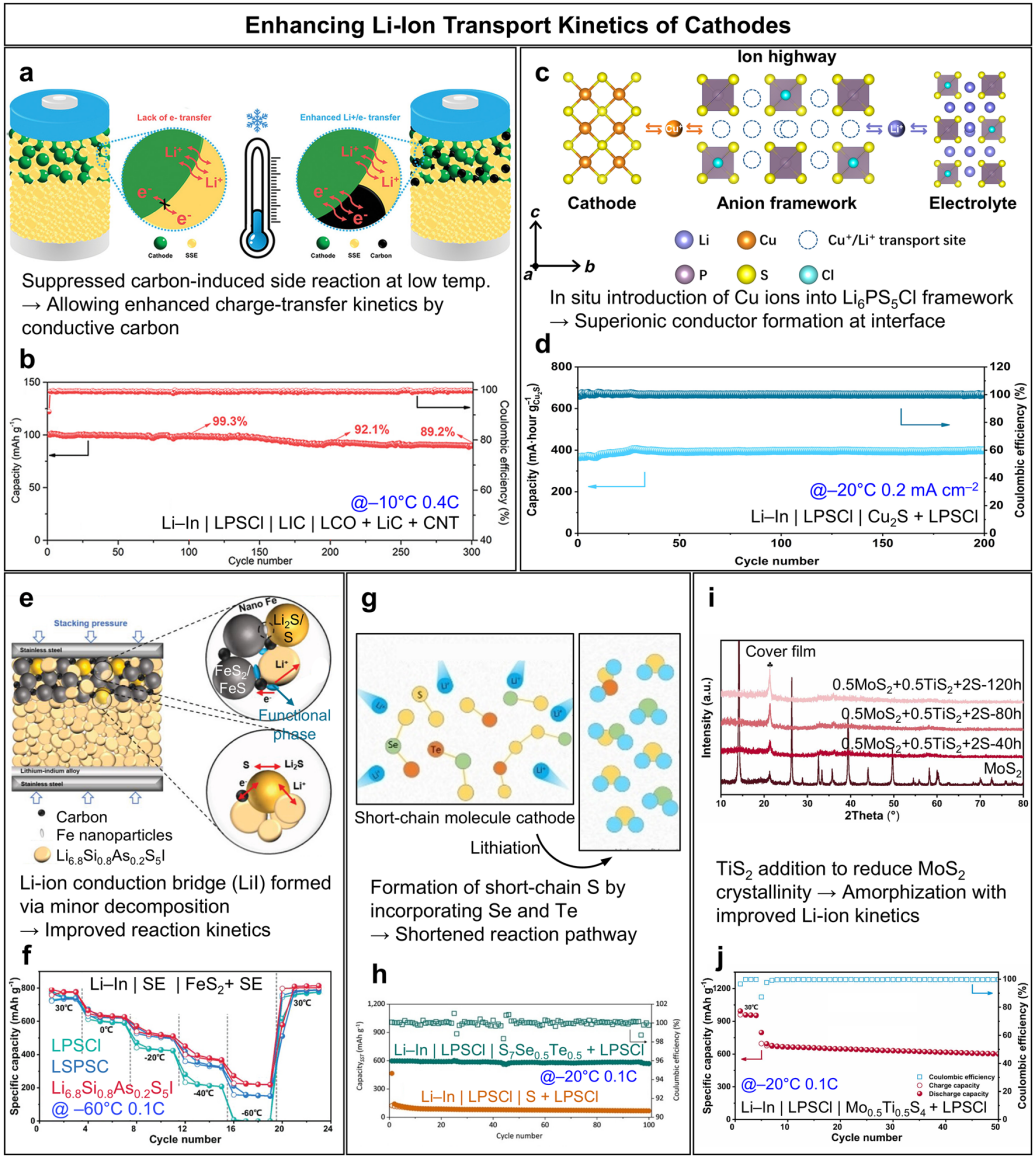
**a** Schematic illustration of Li-ion and electron transport within the composite cathode under low-temperature conditions. (adapted with permission from Deng et al., Copyright 2022, Wiley) [124]. **b** Cycle performance of the Li-In | LPSCl | LIC | LCO + LIC + CNT cell at – 10 °C under 0.4C (adapted with permission from Deng et al., Copyright 2022, Wiley) [124]. **c** Schematic illustration of the ion-conducting pathway between CAMs and SEs, enabled by a Cu^+^/Li^+^ dual-ion conductor (adapted with permission from Yu et al., Copyright 2023, The American Association for the Advancement of Science) [125]. **d** Cycle performance of the Li-In | LPSCl | Cu_2_S + LPSCl cells at – 20 °C under 0.2 mA cm.^−2^ (adapted with permission from Yu et al., Copyright 2023, The American Association for the Advancement of Science) [125]. **e** Schematic illustration of the FeS_2_ ASSB using Li_6.8_Si_0.8_As_0.2_S_5_I (adapted with permission from Lu et al., Copyright 2022, Wiley) [94]. **f** Cycle performance of Li-In | SE | FeS_2_ + SE cells (SE: LPSCl, LSPSC, Li_6.8_Si_0.8_As_0.2_S_5_I) at – 60 °C under 0.1C (adapted with permission from Lu et al., Copyright 2022, Wiley) [94]. **g** Schematic illustration of enhanced cathode utilization enabled by short-chain molecules (adapted with permission from Zhao et al., Copyright 2024, Wiley) [126]. **h** Cycle performance of the Li-In | LPSCl | S + LPSCl and Li-In | LPSCl | S_7_Se_0.5_Te_0.5_ + LPSCl cells at – 20 °C under 0.1C (adapted with permission from Zhao et al., Copyright 2024, Wiley) [126]. **i** XRD patterns of amorphous Mo_0.5_Ti_0.5_S_4_ after ball milling for 40, 80, and 120 h (adapted with permission from Lu et al., Copyright 2023, Elsevier)[127]. **j** Cycle performance of the Li-In | LPSCl | Mo_0.5_Ti_0.5_S_4_ + LPSCl cell at – 20 °C under 0.1C (adapted with permission from Lu et al., Copyright 2023, Elsevier) [127]

At room temperature, carbon additives in the cathode layer, which exhibit excessively high electronic conductivity, tend to promote side reactions with SEs, making moderately conductive carbons more favorable. To mitigate these side reactions, a considerable number of studies exclude carbon additives from the cathode composite. However, under low-temperature conditions, the suppression of side reactions between carbon additives and halide SEs enables the formation of a more stable interface, thereby allowing the use of highly conductive carbon additives to enhance charge-transfer kinetics. Deng et al. demonstrated that incorporating highly conductive carbon nanotubes (CNTs) into LCO- and LIC-based cathode significantly improved low-temperature charge-transfer kinetics and electrochemical performance (Fig. 8a) [124]. CV analysis of the Li–In | LPSCl | LIC | CNT + LIC cell indicated that, at 25 °C, the high electronic conductivity of CNTs facilitated side reactions between the carbon additive and LIC. However, these side reactions were notably suppressed at − 10 and − 30 °C. This temperature-dependent suppression of side reactions enabled the Li–In | LPSCl | LIC | LCO + LIC + CNT cell to deliver a capacity of 100.4 mAh g^−1^ after 300 cycles at − 10 °C with a capacity retention of 89.2%, demonstrating excellent low-temperature electrochemical performance (Fig. 8b). Note that the cathode composition explicitly includes the carbon additive due to its critical role at low temperatures, whereas other studies cited here omit it for simplification.

While cation migration between components or the formation of secondary phases due to decomposition at the interface is generally considered detrimental to cell performance, such interfacial processes can sometimes play a beneficial role in low-temperature conditions by enhancing interfacial kinetics. Yu et al. combined a Cu_2_S CAM with LPSCl, leading to the in situ introduction of Cu-ions into the anion framework of LPSCl [125]. This process formed a superionic conductor at the interface capable of simultaneously transporting both Cu- and Li-ions, thereby enabling a relay transfer process (Fig. 8c). To describe the cathode reaction mechanism, Cu-ions are extracted from the Cu_2_S cathode and migrate into the electrolyte, while Li-ions occupy their vacant sites, allowing for an efficient four-electron transfer. Cu-ion migration was confirmed by SEM and EDS mapping after charging. The resulting interfacial structure significantly enhanced ionic transport kinetics at extreme temperatures by providing fast migration pathways with low-energy barriers and tortuosity. As a result, the Li–In | LPSCl | Cu_2_S + LPSCl cell maintained a constant discharge capacity of approximately 400 mAh g^−1^ over 200 cycles at − 20 °C (Fig. 8d).

Similarly, Lu et al. reported that the Li_6.75_Si_0.75_As_0.25_S_5_I SE exhibits chemical stability with a FeS_2_ composite cathode, and that the slightly formed decomposition by-products can act as functional phases that enhance reaction kinetics, thereby improving low-temperature performance (Fig. 8e) [94]. In detail, while the agglomeration of Fe^0^, a highly reactive conversion intermediate of the FeS_2_ cathode, is a primary cause of performance degradation, finely dispersed Fe^0^ can provide an electron-conducting pathway within the insulating Li_2_S matrix, facilitating the Li_2_S/S conversion. They also demonstrated that this finely dispersed Fe^0^ exhibits superior interfacial stability with Li_6.75_Si_0.75_As_0.25_S_5_I, with a mutual reaction energy of − 0.06 eV per atom—approximately half that of sulfide-based SEs with Fe^0^. Note that the SE composition used in the calculations (Li_6.75_Si_0.75_As_0.25_S_5_I) was slightly different from the composition used in the electrochemical experiments (Li_6.8_Si_0.8_As_0.2_S_5_I) to simplify the computational process. Li_6.8_Si_0.8_As_0.2_S_5_I can undergo slight decomposition during synthesis or battery cycling to form LiI, which serves as an interfacial Li-ion conduction bridge due to its Li-ion exchange capability. These characteristics, combined with the inherently high ionic conductivity of Li_6.8_Si_0.8_As_0.2_S_5_I (10.4 mS cm^−1^ at 25 °C), enabled the FeS_2_-based ASSB to exhibit a greater pseudocapacitive contribution compared to cells using LPSCl or Li_9.5_Si_1.5_P_1.5_S_11_Cl (LSPSC) SEs. This suggests that the electrochemical process is primarily a surface-controlled reaction mechanism with fast reaction kinetics. Consequently, the Li–In | Li_6.8_Si_0.8_As_0.2_S_5_I | FeS₂ + Li_6.8_Si_0.8_As_0.2_S_5_I cell delivered a high discharge capacity of 226.8 mAh g^−1^ even at − 60 °C, which is significantly higher than those using LPSCl (0.3 mAh g^−1^) or LSPSC (154 mAh g^−1^) SEs (Fig. 8f). Cells employing Li_6.8_Si_0.8_As_0.2_S_5_I SEs showed significantly suppressed increases in grain boundary resistance, SEI/CEI Li-ion transport resistance, and charge-transfer resistances upon cooling from 30 to − 60 °C, compared to cells using LPSCl or LSPSC SEs, as confirmed by electrochemical impedance spectroscopy (EIS) and distribution of relaxation times (DRT) analyses.

Introducing multi-chalcogen elements to modulate the local entropy in sulfur (S) cathodes enables the formation of short-chain sulfur species, which can help overcome the sluggish Li-ion kinetics typically associated with long-chain S_8_ molecules at low temperatures. Zhao et al. reported that incorporating selenium (Se) and tellurium (Te) into S cathodes increases the local conformational entropy, thereby stabilizing otherwise unstable short-chain S molecules and shortening the reaction pathway. This effect, which leads to a reduced bond-breaking energy requirement, was revealed by ab initio molecular dynamics (AIMD) simulations (Fig. 8g) [126]. The calculated Gibbs free energy further confirmed that the short-chain molecules exhibit a lower energy barrier for the S reduction process compared to the conventional S_8_ cathode. Benefiting from the enhanced reaction kinetics, the Li–In | LPSCl | S_7_Se_0.5_Te_0.5_ + LPSCl cell delivered an impressive discharge capacity of 559.9 mAh g^−1^ and a capacity retention of 98.4% after 100 cycles at − 20 °C. In contrast, the cell using a conventional S_8_ cathode maintained a much lower capacity of approximately 80 mAh g^−1^ under the same conditions (Fig. 8h).

Controlling the crystallinity of transition-metal sulfide CAMs can significantly enhance their diffusion kinetics. Lu et al. investigated MoS_2_-based transition-metal sulfide CAMs, which possess higher electronic conductivity and improved reaction kinetics compared to S cathodes [127]. However, the rigid crystalline structure of MoS_2_ is unfavorable for Li-ion diffusion. To overcome this limitation, TiS_2_, a material with a low elastic modulus, was introduced during ball milling to promote the amorphization of crystalline MoS_2_. This process resulted in the formation of an amorphous bimetallic polysulfide, Mo_0.5_Ti_0.5_S_4_. As shown in Fig. 8i, the ball-milled Mo_0.5_Ti_0.5_S_4_ sample exhibited no sharp XRD peaks after 40 h of milling, confirming its amorphous nature. To analyze Li-ion transport behavior, DRT analysis was performed on Li–In | LPSCl | MoS_2_ and Li–In | LPSCl | Mo_0.5_Ti_0.5_S_4_ cells down to –60 °C. A significant difference was observed in the peak corresponding to Li-ion diffusion, particularly at lower temperatures, where the Mo_0.5_Ti_0.5_S_4_ cell exhibited a more pronounced improvement. Owing to the improved diffusion kinetics, the Li–In | LPSCl | Mo_0.5_Ti_0.5_S_4_ + LPSCl cell demonstrated excellent low-temperature performance, retaining 86.7% of its initial capacity after 45 cycles at – 20 °C (Fig. 8j).

#### Suppressing Interfacial Degradation

**Fig. 9:**
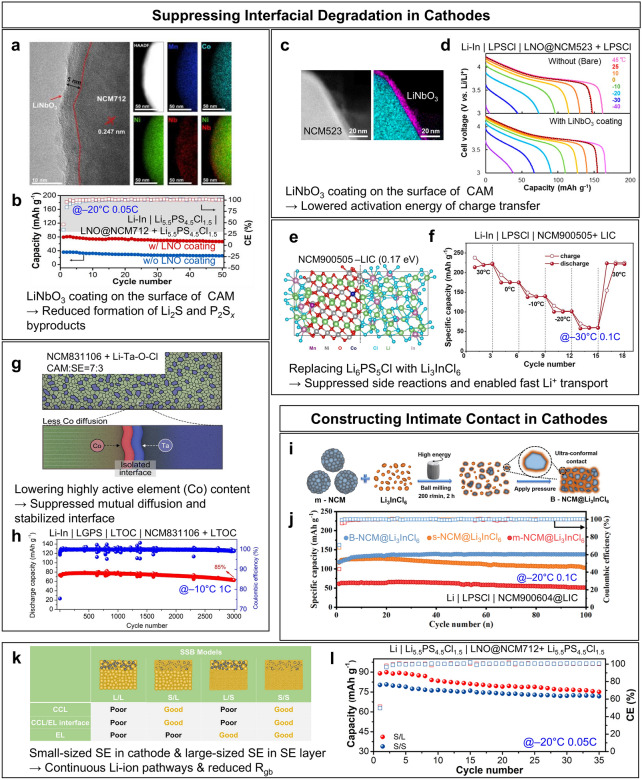
**a** TEM image and corresponding EDS mapping results of LNO@NCM712 (adapted with permission from Peng et al., Copyright 2021, Elsevier) [128]. **b** Cycle performance of the Li-In | Li_5.5_PS_4.5_Cl_1.5_ | LNO@NCM712 + Li_5.5_PS_4.5_Cl_1.5_ cell at – 20 °C under 0.05C (adapted with permission from Peng et al., Copyright 2021, Elsevier) [128]. **c** TEM image and corresponding EDS mapping results of LNO@NCM523 (light blue: Ni, pink: Nb), adapted from Morino et al., Copyright 2021, The Electrochemical Society of Japan[129]. **d** Discharge curves of Li-In | LPSCl | NCM523 + LPSCl and Li-In | LPSCl | LNO@NCM523 + LPSCl cells at various temperatures (45 °C to –40 °C) at 0.1C (adapted from Morino et al., Copyright 2021, The Electrochemical Society of Japan) [129]. **e** Structure of the NCM900505–LIC interface after 10 ps AIMD simulation at 400 K (adapted with permission from Lu et al., Copyright 2024, American Chemical Society)[130]. **f** Temperature-dependent specific capacity of the Li-In | LPSCl | NCM900505 + LIC cell (30 °C to − 30 °C) under 0.1C (adapted with permission from Lu et al., Copyright 2024, American Chemical Society) [130]. **g** Schematic illustration of the interface formation between NCM831106 and the Li-Ta-O-Cl (LTOC) SE (adapted with permission from Zhao et al., Copyright 2024, Royal Society of Chemistry)[131]. **h** Cycle performance of the Li-In | LGPS | LTOC | NCM831106 + LTOC cell at –10 °C under 1C (adapted with permission from Zhao et al., Copyright 2024, Royal Society of Chemistry)[131]. **i** Schematic illustration of the preparation process for B-NCM@Li_3_InCl_6_ (adapted with permission from Zhang et al., Copyright 2023, Royal Society of Chemistry)[132]. **j** Cycle performance of Li | LPSCl | NCM900604@LIC cells (B-, s-, and m-NCM@Li_3_InCl_6_) at – 20 °C under 0.1C (adapted with permission from Zhang et al., Copyright 2023, Royal Society of Chemistry) [132]. **k** Summary of pros and cons for L/L, S/L, L/S, and S/S configurations. (CCL: composite cathode layer; EL: electrolyte layer), adapted with permission from Peng et al., Copyright 2021, Zhengzhou University) [133]. **l** Cycle performance of Li | Li_5.5_PS_4.5_Cl_1.5_ | LNO@NCM712 + Li_5.5_PS_4.5_Cl_1.5_ cells with S/L and S/S configurations at – 20 °C under 0.05C (adapted with permission from Peng et al., Copyright 2021, Zhengzhou University) [133]

Introducing a protection layer on the surface of CAMs can suppress side reactions with sulfide-based SEs and mitigate the increase in charge-transfer resistance, which results in high Li-ion transport across the interface between the CAM and the SE at sub-zero temperatures. Peng et al. introduced a ~ 5 nm-thick LNO coating layer onto the surface of LiNi_0.7_Co_0.1_Mn_0.2_O_2_ (NCM712) (Fig. 9a) [128]. Electrodes with the coating exhibited significantly reduced formation of Li_2_S and P_2_S_*x*_ peaks—both indicative of SE decomposition—after 300 cycles, according to S 2*p* XPS analysis. Due to this suppression of interfacial degradation, the Li–In | Li_5.5_PS_4.5_Cl_1.5_ | LNO@NCM712 + Li_5.5_PS_4.5_Cl_1.5_ cell delivered a much higher initial discharge capacity of 79.3 mAh g^−1^ at − 20 °C, compared to only 35.7 mAh g^−1^ for the uncoated cell. It also maintained over 70 mAh g^−1^ after 50 cycles (Fig. 9b). Similarly, Morino applied a ~ 2–10 nm LNO layer onto LiNi_0.5_Co_0.2_Mn_0.3_O_2_ (NCM523) using a Li–Nb double alkoxide method (Fig. 9c) [129]. While the activation energy of the SE layer resistance remained comparable with and without the coating, that of the charge-transfer resistance associated with Li-ion intercalation/deintercalation into NCM523 was significantly reduced. The Li–In | LPSCl | LNO@NCM523 + LPSCl cell exhibited notably improved discharge capacity over a wide temperature range, from 45 °C down to − 40 °C (Fig. 9d).

Replacing sulfide-based SEs, which are commonly used in cathodes, with electrochemically more stable halide SEs can effectively suppress interfacial side reactions and enhance low-temperature performance. Lu et al. investigated this approach by fabricating Li–In half cells, using LiNi_0.90_Co_0.05_Mn_0.05_O_2_ (NCM900505) as the CAM and either LPSCl or LIC as the SE in the cathode composite [130]. In the NCM900505 + LPSCl cell, an undesired interphase formed by interfacial reactions led to a high interface activation energy of 60.19 kJ mol^−1^, whereas the NCM900505 + LIC cell exhibited a significantly lower value of 25.79 kJ mol^−1^, indicating a more stable interface. Furthermore, the energy barriers for Li atom diffusion at the interfaces were 0.28 and 0.48 eV for NCM900505–Li_2_S and NCM900505–Li_3_PO_4_, respectively—both major by-products formed at the NCM900505/LPSCl interface—while the barrier was only 0.17 eV for NCM900505–LIC, suggesting that LIC effectively suppresses side reactions and enables faster Li transport (Fig. 9e). Electrochemical testing of the Li–In | LPSCl | NCM900505 + LIC cell further demonstrated superior low-temperature performance, retaining 26.9% (57.3 mAh g^−1^) of its 30 °C discharge capacity even at − 30 °C (Fig. 9f). In contrast, the counterpart cell using LPSCl in the cathode composite retained only 2.1% under the same conditions, highlighting the critical role of interfacial stability in low-temperature operation.

Tuning the elemental composition of CAMs can effectively suppress interfacial side reactions with SEs. Zhao et al. investigated the interfacial stability between the lithium tantalum oxychloride (Li-Ta-O-Cl, LTOC) SE and CAMs with varying Co content [131]. In LCO, Co exhibits higher kinetic activity, which promotes mutual diffusion and interaction with Ta from the LTOC SE, resulting in the formation of an unstable interface that continuously grows over time. In contrast, when using LiNi_0.83_Co_0.11_Mn_0.06_O_2_ (NCM831106) with reduced Co content, a passivation layer was formed in situ—presumably consisting of LiTa_3_O_8_, an ion-conducting reaction product—which effectively prevented further decomposition and stabilized the interface (Fig. 9g). High-angle annular dark-field (HAADF) imaging and electron energy loss spectroscopy (EELS) elemental mapping of the LCO/LTOC interface revealed deep Co diffusion across the interface. In contrast, the NCM831106/LTOC interface exhibited a distinct boundary, indicating the suppression of transition-metal diffusion. As a result, the Li–In | LGPS | LTOC | NCM831106 + LTOC cell maintained a capacity of ~ 72 mAh g^−1^ with 85% retention even after 3000 cycles at – 10 °C (Fig. 9h). It is worth noting that NCM523 showed only moderate performance, due to the trade-off between the detrimental effect of Co-induced reactivity and the beneficial formation of a passivation layer.

#### Constructing Intimate Contact Between Components

Through mechanical engineering, ultraconformal interfaces between the CAMs and SEs can be formed, which minimizes solid–solid contact loss. Zhang et al. employed high-energy ball milling with microsized LiNi_0.9_Co_0.06_Mn_0.04_O_2_ (NCM900604, m-NCM) and the halide LIC SE [132]. During this process, the mechanical force fractured the m-NCM particles into submicron-sized NCM (s-NCM), which were uniformly coated with ductile LIC, forming B-NCM@Li_3_InCl_6_ (Fig. 9i). To evaluate Li-ion transport, the Li-ion diffusion coefficient (D_Li⁺_) was calculated via GITT analysis for three types of cathodes: B-NCM@Li_3_InCl_6_, a hand-ground composite of s-NCM and LIC (s-NCM@Li_3_InCl_6_), and a hand-ground composite of m-NCM and LIC (m-NCM@Li_3_InCl_6_). At − 20 °C during the initial charge/discharge, B-NCM@Li_3_InCl_6_ exhibited D_Li⁺_ values of 2.4 × 10^−11^ cm^2^ s^−1^ for both processes, which were higher than those of s-NCM@Li_3_InCl_6_ (1.5 × 10^−11^ cm^2^ s^−1^ for both processes) and m-NCM@Li_3_InCl_6_ (4.6 × 10^−12^ / 1.3 × 10^−12^ cm^2^ s^−1^). This improved Li-ion mobility is attributed to the intimate interfacial contact between the CAMs and SEs. As a result, the Li | LPSCl | B-NCM@Li_3_InCl_6_ cell delivered a discharge capacity exceeding 130 mAh g^−1^ after 100 cycles at − 20 °C, demonstrating excellent low-temperature electrochemical performance (Fig. 9j).

Minimizing the particle size of SEs within the cathode is an effective strategy to maximize the solid–solid contact area and establish continuous Li-ion transport pathways during low-temperature operation. Peng et al. systematically investigated this effect by tuning the particle size of the Li_5.5_PS_4.5_Cl_1.5_ SE (Fig. 9k) [133]. A cell employing large-sized (~ 60 μm) SE particles in the cathode exhibited poor interfacial contact and numerous voids within both the composite cathode layer (CCL) and the CCL/electrolyte layer (EL) interface. In contrast, the use of small-sized (~ 1 μm) SE particles in the cathode resulted in a much denser microstructure in the CCL and at the CCL/EL interface. A similar trend was observed in the EL, where large-sized SE particles led to void formation, while small-sized SE particles produced a densely packed microstructure. The formation of a well-connected ionic conduction pathway was evidenced by the more homogeneous sulfur distribution in the cell containing small-sized SE particles in both the cathode and EL, as confirmed by EDS elemental mapping. Electrochemical performance was evaluated at − 20 °C using Li–In | Li_5.5_PS_4.5_Cl_1.5_ | LNO@NCM712 + Li_5.5_PS_4.5_Cl_1.5_ cells. Two configurations were tested: one with a small-sized SE in the cathode and a large-sized SE in the SE layer (S/L), and the other with a small-sized SE in both layers (S/S). After 35 cycles, both cells retained decent capacities of 75 and 70 mAh g^−1^, respectively (Fig. 9l). Notably, the slightly higher performance of the S/L cell is attributed to the higher ionic conductivity of the large-sized SE in the SE layer, which offers lower grain boundary resistance (R_gb_) compared to the small-sized counterpart (7.0 × 10^−3^ vs. 2.8 × 10^−3^ S cm^−1^).

#### Limitations and Trade-Offs in Cathodes

Strategies developed to mitigate the limitations of cathodes under low-temperature conditions also involve inherent trade-offs. Reducing particle size and introducing conductive coatings are widely adopted to accelerate reaction kinetics and improve charge transfer. However, these approaches increase surface area and interfacial exposure, which can promote undesirable side reactions and structural degradation, particularly during repeated cycling at low temperatures. In contrast, surface protection layers, including artificial interphases and passivation coatings, are effective in stabilizing the CAM–SE interfaces and suppressing undesirable side reactions. Nevertheless, these additional layers can introduce transport limitations, acting as resistive barriers that hinder Li-ion migration and exacerbate polarization when charge-transfer kinetics are already sluggish.

Composite cathode architectures incorporating conductive additives and solid electrolytes are designed to improve ionic and electronic transport. While such designs enhance percolation pathways and reduce kinetic limitations, they inevitably increase the fraction of inactive components, leading to a reduction in overall energy density. Increasing the content of conductive additives improves electronic percolation but can reduce the fraction of solid electrolytes, thereby disrupting continuous Li-ion transport pathways and introducing ionic transport limitations. Beyond energy density considerations, the use of multi-component cathode systems, gradient structures, or nanoscale engineering can improve low-temperature performance but requires more complex synthesis and electrode fabrication processes. These factors can introduce variability in electrode structure and pose challenges for large-scale manufacturing. Such complexity can also hinder reproducibility, making it difficult to achieve consistent electrochemical performance across different batches and cell configurations.

At the materials level, conductive additives are widely used to compensate for the intrinsically low electronic conductivity of CAMs. However, carbon-based conductive additives can induce undesirable side reactions with sulfide SEs through enhanced electron percolation pathways, leading to interfacial instability. Conductive additives exist in various forms, such as nanotubes, nanorods, and spherical particles, each with distinct surface areas and electronic conductivities. While high-surface-area additives can improve electronic connectivity, they can also increase interfacial reactivity. Their interaction with the CAM and the SE can therefore lead to different interfacial behaviors depending on the specific material combination and electrolyte chemistry. As a result, the selection and content of conductive additives require careful optimization, considering both electronic transport enhancement and interfacial stability.

Cathode performance at low temperatures reflects the interaction between reaction kinetics, interfacial stability, and electrode architecture, such that improvements in one aspect often introduce trade-offs in others. As a result, enhancing cathode performance under sub-zero conditions requires balancing electrochemical activity with structural and practical constraints.

### Anode

The anode critically influences the low-temperature performance of ASSBs, as Li-ion transport and interfacial charge transfer at the anode–SE interface are strongly temperature-dependent. At sub-zero temperatures, sluggish Li-ion diffusion, interfacial instability, and poor electronic connectivity often lead to uneven Li deposition, dendritic growth, and severe polarization. Notably, the formation and propagation of Li dendrites become more pronounced under low-temperature conditions due to localized Li-ion flux and sluggish kinetics. A smaller nucleus size and higher nucleation density further facilitate the growth of needle-like dendritic structures, thereby increasing the risk of internal short circuits and cell failure. Consequently, ensuring both interfacial stability and efficient ion transport across the anode–SE interface is essential to enable reliable ASSB operation under low-temperature conditions.

To address these issues, recent efforts have focused on developing advanced anode designs that enable uniform Li-ion transport and stable electrochemical interfaces. In particular, mitigating dendrite formation while maintaining fast Li-ion transport remains a key challenge in low-temperature battery systems. Recent advances have mainly focused on three major strategies to improve the low-temperature performance of ASSB anodes. These include constructing intimate interfacial contact between layers, suppressing interfacial degradation at the anode–SE interface, and introducing conductive pathways to enhance charge transport properties. These strategies aim not only to improve electrochemical performance but also to regulate Li deposition behavior and suppress dendrite growth under sub-zero conditions. The following sections summarize representative approaches based on these strategies and highlight their roles in improving the low-temperature performance of ASSB anodes.

#### Enhancing Transport Kinetics

**Fig. 10:**
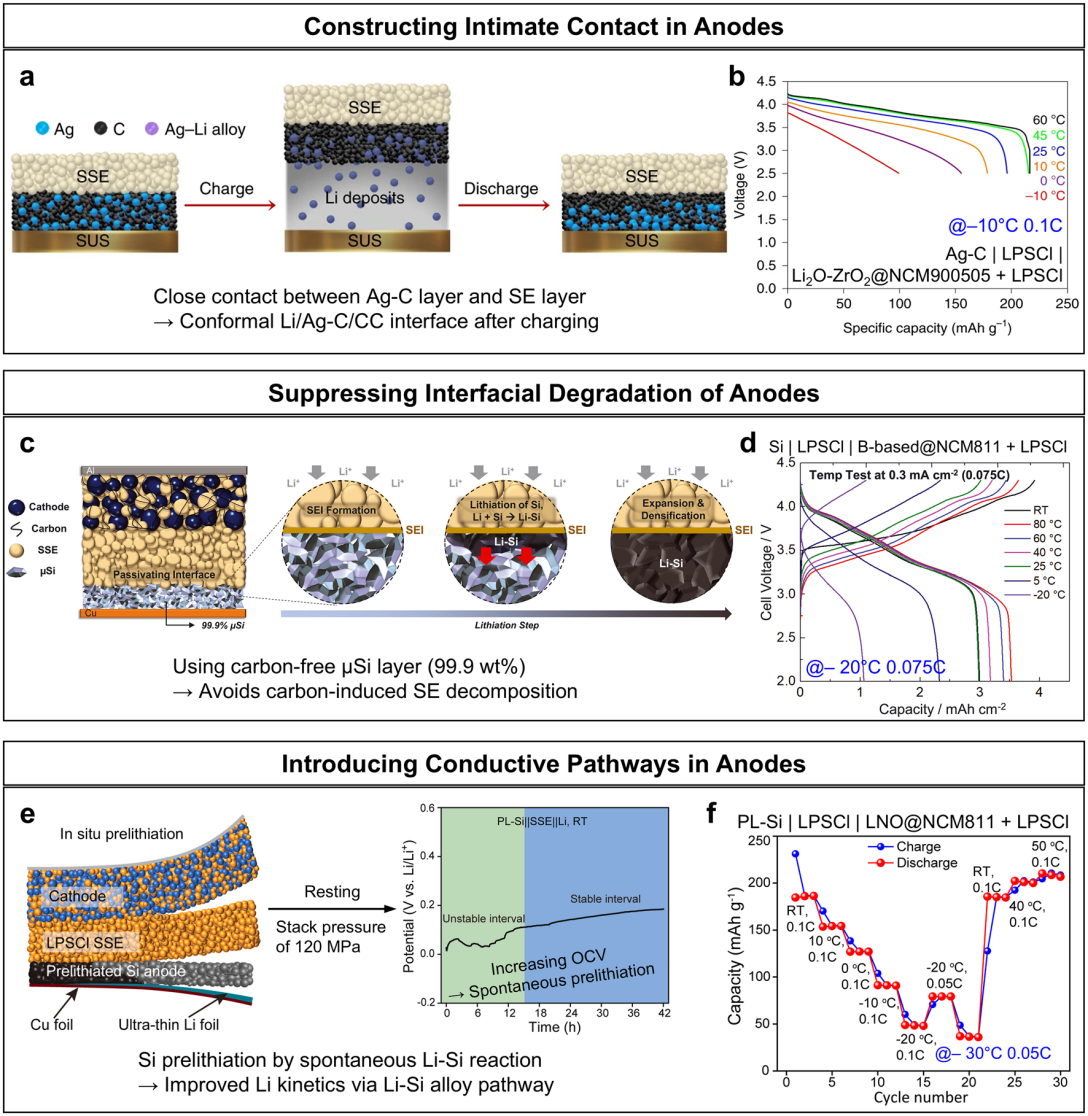
**a** Schematic illustration of Li plating and stripping on the current collector with Ag-C layer during cell operation. (adapted with permission from Lee et al., Copyright 2020, Springer Nature) [136]. **b** Discharge curves of the Ag-C | LPSCl | Li_2_O-ZrO_2_@NCM900505 + LPSCl cell at various temperatures (60 °C to – 10 °C) at 0.1C (adapted with permission from Lee et al., Copyright 2020, Springer Nature). The charging temperature was fixed at 60 °C [136]. **c** Schematic illustration of the cell with μSi anode during lithiation (adapted with permission from Tan et al., Copyright 2021, The American Association for the Advancement of Science) [135]. **d** Charge–discharge curves of the Si | LPSCl | B-based@NCM811 + LPSCl cell at various temperatures (80 °C to − 20 °C) at 0.075C (adapted with permission from Tan et al., Copyright 2021, The American Association for the Advancement of Science) [135]. **e** Schematic illustration of the in situ prelithiation strategy of the Si anode (left) and open-circuit voltage profile of the Li | SE | PL-Si cell during 42 h rest (right), adapted with permission from Fan et al., Copyright 2023, Elsevier[134]. **f** Temperature-dependent specific capacity of the PL-Si | LPSCl | LNO@NCM811 + LPSCl cell (50 °C to − 30 °C) under 0.05C and 0.1C (adapted with permission from Fan et al., Copyright 2023, Elsevier) [134]

In conventional anode materials, such as graphite and silicon, large interfacial resistance at the anode–SE interface further exacerbates kinetic limitations, leading to sluggish Li-ion transport and increased polarization. These challenges become more severe at sub-zero temperatures, where both bulk ionic conductivity and interfacial charge transfer are strongly hindered. The prelithiation method has been investigated to address the intrinsically sluggish kinetics of Si anodes. Pristine Si, as a semiconductor, has limited electronic conductivity and extremely low Li-ion conductivity. In contrast, lithiated Si exhibits significantly improved electronic and Li-ion conductivities, enabling much faster Li-ion transport. Based on this principle, Fan et al. introduced an ultrathin Li foil (10 μm) between the Cu current collector and the Si anode [134]. Under stack pressure, an increase in open-circuit voltage was observed, indicating a spontaneous reaction between Li and Si (Fig. 10a). After 24 h of rest under 120 MPa, the Li foil fully reacted with the Si anode to form prelithiated Si (PL-Si, with a Li_0.85_Si composition). Compared to the cell with the conventional Si anode, the PL-Si | LPSCl | LNO@NCM811 + LPSCl cell exhibited narrower voltage gaps between anodic and cathodic peaks and higher peak intensities in the dQ dV^−1^ profiles, confirming enhanced Li kinetics. Electrochemical tests of the PL-Si | LPSCl | LNO@NCM811 + LPSCl cell demonstrated high discharge capacities of 79.2 and 36.4 mAh g^−1^ at − 20 and − 30 °C, respectively (Fig. 10b). These results demonstrate that improving both electronic and ionic transport within the anode can effectively mitigate kinetic limitations, particularly under low-temperature conditions where transport processes are inherently constrained.

#### Suppressing Interfacial Degradation

In sulfide-based SEs, high ionic conductivity is often accompanied by limited electrochemical stability, particularly in contact with electronically conductive carbon materials. The presence of carbon can facilitate electron transfer at the interface, thereby accelerating undesirable side reactions of the sulfide electrolyte. Suppressing interfacial decomposition at the anode/SE interface can be achieved by removing carbon additives, which are known to promote SE degradation. Tan et al. reported that pure microsilicon (μSi) anodes exhibit excellent electrochemical performance in ASSBs over a wide temperature range [135]. The inclusion of carbon in the Si + LPSCl + carbon (20 wt%) composite anode leads to electrochemical decomposition of the SE near 2.5 V, even before reaching the lithiation potential, as revealed by the voltage profiles of full cells using LiNi_0.8_Co_0.1_Mn_0.1_O_2_ (NCM811) cathodes. This undesirable decomposition originates from carbon-induced electron percolation pathways, which induce reductive decomposition of the solid electrolyte at the anode–SE interface. Moreover, the promoted formation of highly passivating Li_2_S species was confirmed by S 2*p* and Li 1*s* XPS analyses. To suppress such decomposition, a carbon-free anode architecture using 99.9 wt% μSi was designed, minimizing SE side reactions (Fig. 10c). Electrochemical evaluation of the μSi | LPSCl | NCM811 + LPSCl cell showed that ~ 35% of the room-temperature capacity was retained even at − 20 °C (Fig. 10d). These results highlight that eliminating electronically conductive additives can effectively suppress interfacial side reactions and stabilize the anode–SE interface, although such strategies may introduce trade-offs in electronic conductivity within the electrode.

#### Constructing Intimate Contact Between Layers

In Li metal anode systems, the intrinsic mismatch in mechanical properties between Li metal and the SEs often leads to the formation of interfacial voids during cycling, particularly during Li stripping. These voids disrupt physical contact, induce current constriction at limited contact points, and ultimately result in non-uniform Li deposition and accelerated interfacial degradation. Conventional interlayer or coating strategies can improve initial interfacial contact, but they often suffer from a critical limitation in that Li tends to deposit on top of the interlayer, leading to progressive interfacial separation and limited long-term stability. To overcome this limitation and ensure stable solid–solid contact with the SE layer, functional interlayers have been introduced. Lee et al. implemented a silver–carbon (Ag–C) composite layer, which also serves as a Li-regulating interlayer, on top of the current collector (CC) (Fig. 10e) [136]. The nanoporous Ag–C layer maintained close contact with the LPSCl layer even immediately after cell assembly. Moreover, the application of Ag enhances the conductivity and decreases the nucleation energy of Li metal, thereby facilitating dense and uniform Li deposition after the initial charging. Importantly, unlike conventional interlayers, Li deposition occurs preferentially beneath the Ag–C layer at the current collector interface, enabled by Ag–Li alloy formation and lithiophilic characteristics. This allows Li to be reversibly plated and stripped without accumulating within or above the interlayer, thereby mitigating void formation and maintaining stable interfacial contact. Cross-sectional SEM images after several cycles further confirmed the absence of residual Li deposits and dendritic growth within the Ag–C layer. In contrast, in the absence of the interlayer, the poor solid–solid contact resulted in current localization at limited interfacial sites, leading to inhomogeneous Li deposition and insufficient Li/SE contact. A 0.6 Ah Ag–C | LPSCl | Li_2_O–ZrO_2_@NCM900505 + LPSCl pouch cell retained over 40% of its discharge capacity at − 10 °C compared to 60 °C (Fig. 10f). Similarly, Wu et al. demonstrated that while significant voids were observed at the Li/LPSCl interface immediately after cell assembly, introducing an Ag–C interlayer between the Li and SE resulted in well-formed interfacial mechanical contact. This led to a substantial reduction in interfacial resistance and promoted homogeneous current distribution [137].

#### Limitations and Trade-Offs in Anodes

Various approaches to address the limitations of anodes under low-temperature conditions are associated with coupled trade-offs in Li deposition behavior, interfacial stability, and transport properties. In anode systems, these factors are particularly critical as they are closely linked to dendrite formation under these conditions. To overcome sluggish Li-ion transport at low temperatures, strategies that enhance electronic and ionic conductivity are often employed. However, careful control over the spatial distribution of conductivity is required, as localized enhancement in electronic or ionic pathways can induce uneven Li-ion flux and current density, thereby promoting non-uniform Li deposition and dendritic growth under sub-zero conditions.

Protective layers and artificial interphases are also commonly introduced to suppress interfacial side reactions and stabilize the anode–electrolyte interface. While these layers can mitigate electrolyte decomposition, they may also hinder Li-ion transport or increase interfacial resistance, which becomes more pronounced at low temperatures. The incorporation of such layers can increase the fraction of inactive components, leading to a reduction in overall energy density. Host-assisted designs, such as porous frameworks and lithiophilic scaffolds, are widely adopted to improve interfacial contact and regulate Li nucleation. While these approaches can enhance deposition uniformity, they often increase structural complexity and the fraction of inactive components, leading to a reduction in energy density. Non-uniform Li distribution can still arise during prolonged cycling. Overall, Li deposition behavior, interfacial stability, and transport properties are strongly coupled in anode systems, and improvements in one aspect often introduce new constraints in others. As a result, mitigating dendrite formation while maintaining stable performance remains a central challenge for low-temperature ASSBs.

## Future Perspective

**Fig. 11:**
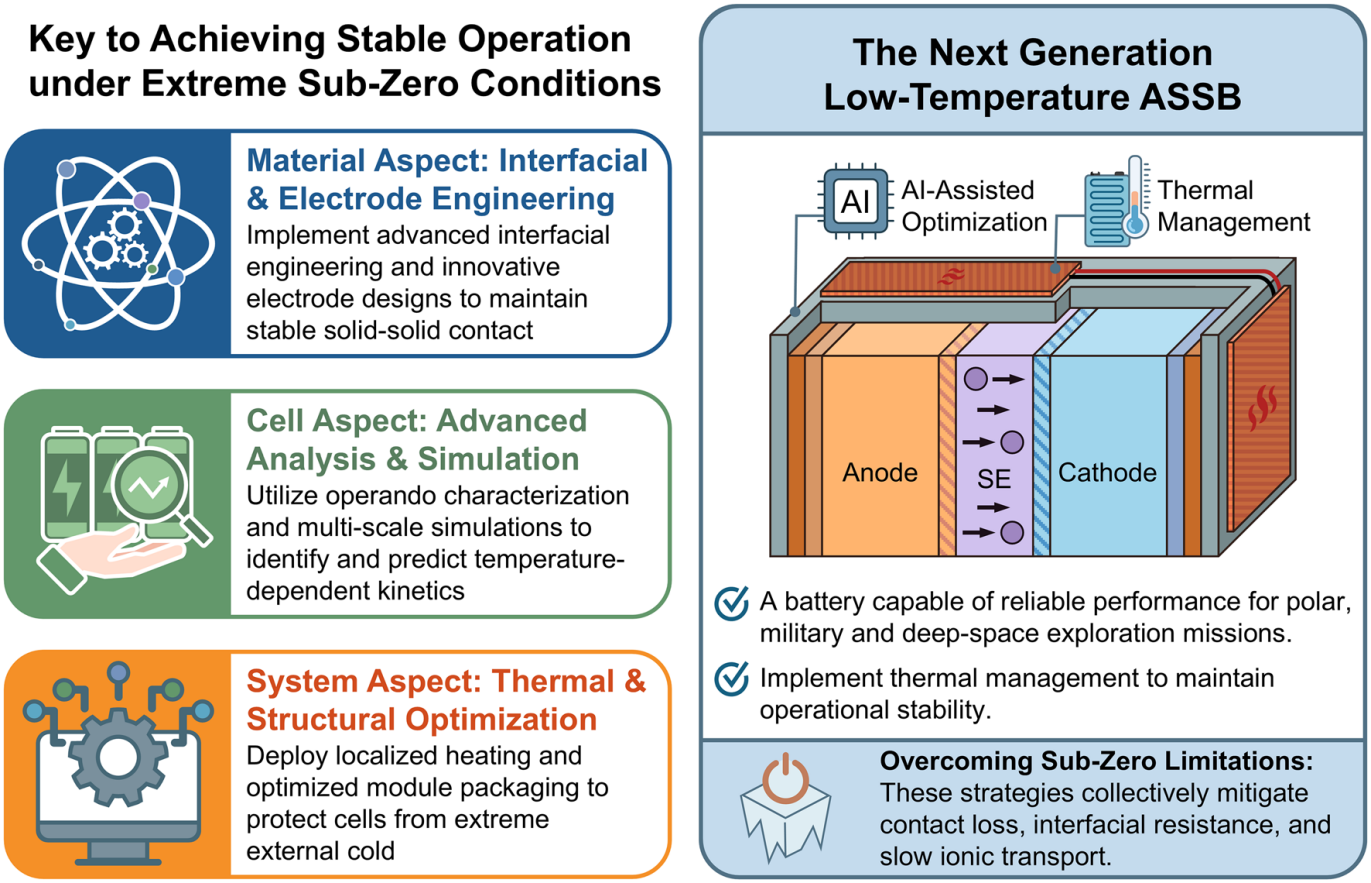
Key strategies from the material to the system level for stable low-temperature operation

With the rapid expansion of energy storage applications into harsh environments, the demand for batteries capable of reliable operation at sub-zero temperatures has never been greater. ASSBs have significant potential due to their non-flammable SEs, improved thermal stability, and enhanced safety compared to conventional LIBs. However, despite notable advancements, achieving stable performance under extremely low-temperature conditions remains a critical challenge. As shown in Fig. 11, future research must take a comprehensive approach that integrates interfacial engineering, electrode architecture optimization, mechanistic understanding, and system-level strategies to overcome the intrinsic Li-ion transport and stability limitations of ASSBs at low temperatures.

At room temperature, ionic transport properties vary significantly depending on the material system. Transition-metal oxide cathodes typically exhibit Li-ion diffusivity in the range of 10^−14^ to 10^−9^ cm^2^ s^−1^, while graphite anodes show relatively higher values on the order of 10^−7^ to 10^−6^ cm^2^ s^−1^ at room temperature [138–141]. Solid electrolytes exhibit a wide range of ionic conductivities depending on their material type. Sulfide-based solid electrolytes typically show high ionic conductivities on the order of 10 ^−2^ S cm^−1^, whereas oxide- and polymer-based systems generally exhibit lower values around 10^−4^ S cm^−1^ [142–146]. However, under low-temperature conditions, ionic transport in all these systems is significantly suppressed due to reduced ion mobility. Therefore, achieving ionic transport properties that approach those at room temperature, or at least maintaining comparable orders of magnitude, remains a critical challenge for enabling reliable low-temperature ASSB operation.

One of the most critical challenges in low-temperature ASSBs lies at the solid–solid interfaces, where poor physical contact, interfacial side reactions, and unstable interphases collectively hinder Li-ion transport. At sub-zero temperatures, thermal contraction and mechanical mismatch exacerbate contact loss, leading to non-uniform Li-ion flux. Unintended interfacial side reactions can block Li-ion transport pathways and increase interfacial resistance, thereby accelerating overall cell degradation. Future research should focus on advanced interfacial engineering strategies, such as the introduction of artificial buffer layers, surface modification techniques, and optimized electrode composites, to maintain robust and stable solid–solid contacts under harsh conditions.

In addition to interfacial stability, the design of electrode architecture plays a critical role in enhancing low-temperature performance. High-loading electrodes suffer from severe Li-ion transport limitations at sub-zero conditions, resulting in increased polarization and capacity fade. Innovative electrode design strategies are expected to shorten Li-ion diffusion paths and improve interfacial kinetics. Integrating mechanical flexibility into electrode structures could further mitigate microcracks and contact loss during low-temperature cycling, ultimately enhancing long-term durability. However, these design strategies often require increased fractions of inactive components and more complex architectures, highlighting an inherent trade-off between performance enhancement and practical implementation.

Despite significant progress, the fundamental mechanisms driving performance degradation in ASSBs at low temperatures are not yet fully understood. In particular, a systematic understanding of how these processes evolve and interact under sub-zero conditions is still lacking, limiting the rational design of high-performance ASSBs. The relative contributions of bulk ionic conduction, interfacial charge transfer, and active material diffusion still remain unclear. Therefore, more comprehensive mechanistic studies are urgently required to better understand the temperature-dependent rate-determining steps and failure mechanisms in ASSBs. Advanced in situ and operando characterization techniques, combined with cryogenic electrochemical analyses, are critical for identifying the temperature-dependent rate-determining steps. In parallel, theoretical calculations and multi-scale simulations, such as first-principles calculations, 3D modeling, and AI-assisted optimization, will play a crucial role in predicting Li-ion dynamics and guiding the rational design of high-performance ASSBs for extreme environments.

Beyond material- and cell-level improvements, system-level strategies are key to achieving stable operation under extreme sub-zero conditions. Advanced thermal management solutions, such as localized heating systems and integrated insulation layers, could help maintain cell temperature without compromising energy density. Nevertheless, these additional components tend to increase system complexity, introducing further trade-offs at the cell and system levels. Optimized cell and module designs, including advanced packaging architectures and improved electrode stacking strategies, could further enhance the low-temperature performance and safety of ASSBs in extreme environments.

A critical challenge moving forward is to balance low-temperature performance with practical considerations, such as energy density, cost, and manufacturability. Many of the strategies discussed, including interfacial layers, composite electrode designs, and increased fractions of solid electrolytes or conductive additives, tend to raise the proportion of inactive materials within the cell. As a result, gravimetric and volumetric energy densities are reduced, while material and processing complexity increases. Future efforts should therefore focus on minimizing these trade-offs through intrinsically high-performance materials and simplified architectures, including solid electrolytes with both high ionic conductivity and mechanical robustness, electrodes with fast Li-ion transport kinetics, and interfaces that remain stable without excessive modification. Incorporating scalable fabrication methods and material compatibility from the early design stage will be essential to achieve a balanced optimization between low-temperature capability and overall battery performance.

The continuous advancement of ASSBs holds the key to unlocking reliable energy storage for the most demanding low-temperature applications. While individual strategies have shown promise, their isolated implementation is unlikely to deliver practical performance. Instead, future progress will depend on the synergistic integration of materials design, interface engineering, electrode architecture, and system-level strategies, where these approaches are not treated independently but function cooperatively across multiple length scales. Establishing such an integrated framework will be essential to overcome the coupled limitations of ion transport, interfacial stability, and mechanical integrity under sub-zero conditions. Success in this direction will not only enable ASSBs to meet the requirements of polar expeditions, military missions, and deep-space exploration, but also accelerate the transition toward safer, sustainable, and high-performance energy storage technologies.
